# SciPhy: A Bayesian phylogenetic framework using sequential genetic lineage tracing data

**DOI:** 10.1038/s41467-026-73377-6

**Published:** 2026-06-10

**Authors:** Sophie Seidel, Antoine Zwaans, Samuel Regalado, Junhong Choi, Jay Shendure, Tanja Stadler

**Affiliations:** 1 Department of Biosystems Science and Engineering, ETH Zürich, Basel, Switzerland; 2 Swiss Institute of Bioinformatics, Lausanne, Switzerland; 3 Department of Genome Sciences, University of Washington, Seattle, WA USA; 4 Developmental Biology Program, Memorial Sloan Kettering Cancer Center, New York, NY USA; 5 Howard Hughes Medical Institute, Seattle, WA USA; 6 Seattle Hub for Synthetic Biology, Seattle, WA USA; 7 Brotman Bay Institute for Precision Medicine, Seattle, WA USA; 8 Allen Discovery Center for Cell Lineage Tracing, Seattle, WA USA

**Keywords:** Cell division, Organogenesis, Phylogeny, Genomics

## Abstract

CRISPR-based lineage tracing offers a promising avenue to decipher single-cell lineage trees, especially in organisms not amenable to microscopy. Sequential genome editing records not only genetic edits but also the order in which they occur. To leverage this enriched information, we introduce SciPhy, a simulation and inference tool implemented in BEAST 2. SciPhy utilizes a Bayesian phylogenetic approach to jointly estimate time-scaled phylogenies and cell population parameters. After validation on simulated data, we use simulated and real data from a monoclonal cell culture to benchmark SciPhy against existing methods and find that it consistently reconstructs more accurate phylogenies. Compared to UPGMA, SciPhy additionally reports uncertainty and proliferation rates. Our second example applies SciPhy to murine gastruloids, demonstrating its ability to model time-varying population dynamics in early development. Together, these results establish a phylodynamic framework for the quantitative analysis of lineage tracing data. SciPhy’s codebase is publicly available at https://github.com/azwaans/SciPhy.

## Abstract (web summary)

Sequential CRISPR edits hold great potential for reconstructing lineage trees, but current methods largely ignore edit order. Here, the authors develop a Bayesian framework that infers timed phylogenies and captures shifting proliferation dynamics in gastruloids.

## Introduction

The interplay between cell division, cell death, and differentiation is at the core of the development of all multicellular organisms. Tracing cell populations and their lineage relationships via DNA-based molecular recording has recently emerged as a powerful approach to study this complex process^1^. Among the technologies developed for lineage recording, CRISPR–Cas9-based approaches stand out for generating stochastic, heritable indels in genetic barcodes during development and proliferation^2–8^. However, early approaches relying on *unordered indels* combined with a short recording duration limit the reconstruction of highly resolved cell lineage trees (see Glossary, Table 1)^9,10^.

**Table 1:** Glossary: Translation of phylogenetic terminology to cell biology terminology

Phylogenetic terms	Cell biology terms
Tree topology	Lineage tree
Clock rate	Edit insertion rate by prime editor (e.g., per hour)
Bifurcation, branching point	Cell division event
Branching time	Cell division time
Time-scaled tree	Lineage tree where branches are scaled in real time
Monophyletic	Monoclonal

Recent work^9,10^ addresses some of these limitations by introducing prime-editing-based recording systems that enable lineage tracing over longer time periods. These methods rely on *ordered insertions* of nucleotide sequences at target sites mediated by a prime editor and prime editing guide RNA (pegRNA)^11^. Each insertion at a target site deactivates the current site and activates the next for subsequent editing. This ensures irreversible edit accumulation and records the order of edits within the target. These new lineage tracing technologies have the potential to enable the reconstruction of accurate, highly resolved single-cell lineage trees.

To date, lineage tree reconstruction using such data^9,10^ has relied on UPGMA (Unweighted Pair Group Method with Arithmetic Mean^12^) in tandem with custom distance metrics. This approach is agnostic to specific properties of the editing process, such as variable propensities for introducing particular inserts and variable edit insertion rates (see “Glossary”, Table 1). UPGMA, being a distance-based clustering method, only leverages pairwise distances between cells, ignoring all higher-order information^13^. In contrast, Bayesian phylogenetic inference methods for unordered edit lineage tracing data^14^ enable the estimation of lineage trees with cell division times (see “Glossary”, Table 1) by mechanistically modeling barcode generation. This approach was shown to provide accurate lineage reconstruction, attributed to explicitly incorporating a priori knowledge of the editing process and the experimental conditions under which traced cell populations were studied.

In this article, we derive a mechanistic model of sequential insertion-based editing (ordered insertions) for lineage tracing data. This model forms the basis of our new inference framework, ’SciPhy’ **S**equential **c**as9 **i**nsertion-based **Phy**logenetics, implemented in the Bayesian inference software BEAST 2^15^. BEAST 2 uses a Markov Chain Monte Carlo framework to jointly estimate time-scaled lineage trees (see “Glossary”, Table 1) and editing dynamics. This integration further makes phylodynamic analyses of single-cell populations directly accessible to the community.

We showcase the versatility of our validated framework with a comprehensive analysis of two lineage tracing datasets: the first derived from a monoclonal (see “Glossary”, Table 1) HEK293T culture^10^ and the second from the in vitro differentiation of a single mouse embryonic stem cell (mESC) into a multicellular gastruloid, a protocol that models aspects of early mammalian development. Our analyses reveal that complex editing dynamics govern the recording process in both datasets and estimate the growth rates of the cell populations over time. We observe significant differences between our lineage tree estimates and those obtained with UPGMA, underscoring the impact of the reconstruction method on the inferred cellular relationships and growth dynamics.

## Results

**Fig. 1:**
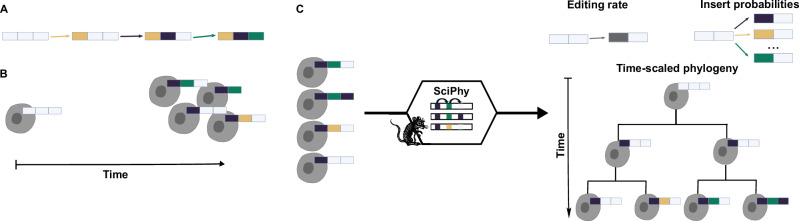
SciPhy is a framework for phylogenetic and phylodynamic analysis of sequential edit lineage tracing data. It implements a mechanistic model of sequential editing-based recording systems (**A**), which enables their use for Bayesian phylodynamic inference in BEAST 2. These alignments are obtained following typical pipelines of genetic lineage tracing experiments: cells that harbor copies of a recording barcode, or ’DNA Tape’, accumulate insertions during growth (**B**). At the end of these experiments, single-cell sequencing generates barcode alignments (**C**), from which SciPhy can infer parameters of the recording system: the rate and probability at which different insertions are acquired.

We introduced a mechanistic editing model and likelihood calculation for sequential lineage tracing data as the core component of SciPhy (Fig. 1). This model assumes that stably integrated barcodes accumulate edits sequentially and irreversibly at a constant Cas9 nicking rate. Subsequent pegRNA-mediated insertions occur with varying probabilities. We derived a phylogenetic likelihood to calculate the probability of observing a barcode alignment given a phylogeny and SciPhy parameters. Details of the model, its parameterization, and Bayesian inference implementation in BEAST 2 are in “Methods”.

### In-silico validation

We validated SciPhy using well-calibrated simulations^16^ to ensure that it draws samples from the correct posterior distribution. This process involved simulating trees and editing barcodes for each cell, which were then used as input data to estimate both the lineage tree and the editing parameters under the SciPhy model. Editing parameters were drawn from distributions representing realistic editing dynamics (Table 2). The same distributions were also used as priors in the inference (see “Methods” for details). Correct tree inference was assessed through three key summary statistics of the inferred lineage trees: tree height, tree length, and tree balance, which collectively cover the properties of branch lengths and tree shape.

**Table 2:** Distributions for the editing model parameters used in the validation

Parameter name	Symbol	Distribution	95% HDI
Clock rate	$r$	Log-normal ($\mu=-2,\sigma=0.5$)	[3.5e-2, 3.1e-1]
Edit probabilities	${f}_{k}$	Dirichlet ($\alpha=1.5$)	[1.9e-4, 1.9e-1]

We calculated the coverage for each editing model parameter and tree summary statistic, defined as the fraction of datasets for which SciPhy inferred a 95% highest posterior density (HPD) interval containing the true (simulation) parameter. The coverages for all parameters fall within the expected range^17^ (see Supplementary Table 2 for values), demonstrating that our method is correctly implemented.

**Fig. 2:**
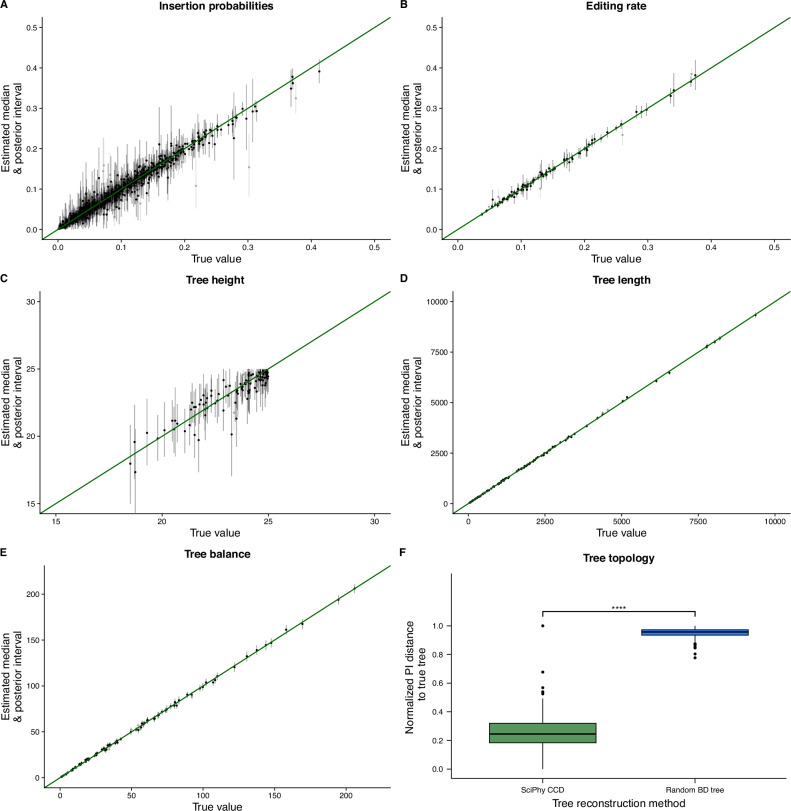
Validation results. We report the results of the in-silico validation for the insertion probabilities (**A**), editing rate (**B**), tree height (**C**), tree length (**D**), B1 tree balance index (**E**), and tree topology (**F**) on N = 100 independent simulations. In all panels (**A**–**E**), dots represent the posterior median, and vertical error bars denote the 95% highest posterior density (HPD) interval. Solid black dots indicate successful recovery (true value within the 95% HPD), while gray dots indicate recovery failure. The dashed green line represents the performance of an ideal estimator. Panel **F**, shows a comparison of topological distances between the ground truth simulated trees and trees reconstructed via SciPhy (SciPhy CCD, green) or simulated under a birth-death model (Random BD tree, blue). Distances are measured using the normalized Phylogenetic Information (PI) metric. Box plots indicate the median (center line), the 25th and 75th percentiles (bounds of box), and the minima and maxima (whiskers), with outliers plotted as points. Statistical significance was assessed using a two-sided paired *t*-test with Bonferroni correction for multiple comparisons. Topologies for which the normalized Phylogenetic Information (PI) distance could not be calculated were excluded from the analysis, resulting in a final sample size of *n* = 96 (*t*(95) = 53.9, adjusted P = 4.91e − 73, mean difference = 0.688, 95% CI of the difference = [0.663, 0.714], Cohen’s *d* = 5.50). Source data are provided as a Source Data file.

To evaluate the inference power across a range of parameter values, we plotted the simulated (true) parameters against the estimated parameters (Fig. 2A–E). The correlations for the editing rate and the insertion probabilities (with a Pearson’s *R* value of 0.99 and 0.98, respectively, see Supplementary Table 2 for 95% confidence intervals) confirm that SciPhy extracts meaningful signal from the data. Likewise, a high correlation for all three tree summary statistics is achieved (for tree height, tree length, and tree balance, Pearson’s R of 0.90, 0.99, and 0.99, respectively), showing that signal for these features is also derived from the data.

We also assess SciPhy’s ability to extract signal for lineage relationships from the data by comparing topological distances (using the Phylogenetic Information (PI) distance^18^) between random trees obtained by simulations under a simple birth-death model as well as SciPhy’s tree estimates (summarized as point estimates with a conditional clade distribution algorithm (CCD)^19^) and the true simulated topologies. SciPhy consistently and significantly recovers topologies closer to the ground truth, thus confirming its ability to learn lineage relationships from the data (Fig. 2F).

Finally, we evaluate SciPhy’s robustness to two major sources of missing data found in lineage tracing datasets, namely, transgene silencing and sequencing dropout. To do so, we simulate alignments under varying levels of heritable tape loss (modeling transgene silencing) and loss upon sampling (modeling sequencing dropout), which both result in incomplete recovery of tape sequences per cell (see “Methods” for a description of this simulation process). Trees reconstructed using incomplete barcode alignments are farther from the ground truth tree than those that use complete ones as input (see Supplementary Fig. 23, top and 24), considering both the tree topology alone (using the PI distance) and branch length estimation (using the weighted Robinson-Foulds (wRF) distance). We find that the PI distance for tree topology is particularly sensitive to tape loss and the resulting alignment sparsity (see Supplementary Figs. 23, bottom left and 24, left), while the wRF metric, considering both topology and branch lengths, results in a weaker increase in distance as tape loss probability increases.

### Phylodynamic analysis of monoclonal expansion

**Fig. 3:**
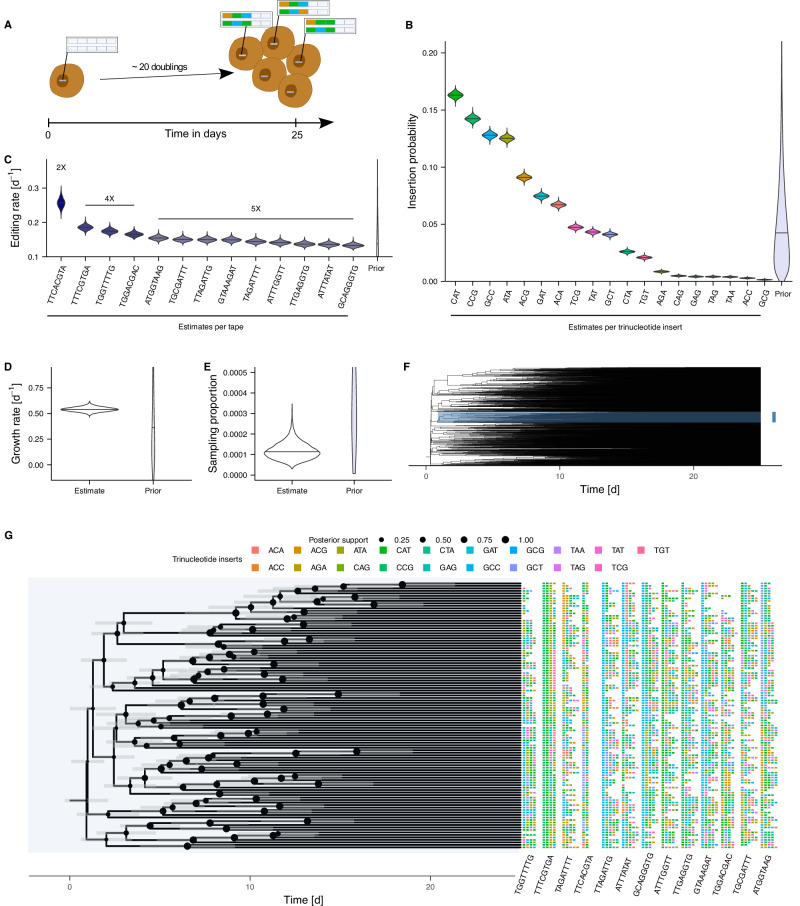
SciPhy captures complex editing dynamics and infers developmental parameters. **A** We apply SciPhy to a dataset of 1000 tape sequences sampled from a cell population generated by a 25-day monoclonal expansion of a single HEK293T progenitor cell harboring the DNA Typewriter recording system (*n* = 1). We report posterior distributions and medians (center lines) of the editing parameters estimated under the SciPhy model, namely the insertion probabilities (**B**) and the editing rates per day per tape (**C**). Editing rates for truncated tapes are colored with darker shades and annotated with the corresponding number of sites (respectively 2, 4, and 5 sites). We report posterior distributions and medians (center lines) of the cell population dynamics estimated under the birth-death-sampling model, namely the growth rate in (**D**) and the sampling proportion in (**E**). In **F**, we plot the time-scaled conditional clade distribution (CCD) summary tree for the 1000 sampled cells and highlight a clade (blue) that is shown enlarged in (**G**). **G** We show a sub-clade of the CCD tree and highlight the posterior support in the node placement as the node thickness and the uncertainty in the cell division times as gray shadings denoting their 95% highest posterior density (HPD) intervals. We plot the DNA Typewriter tape alignment facing the tree tips. Source data are provided as a Source Data file.

We analyze a dataset where DNA Typewriter was used to trace lineage in a 25-day monoclonal expansion of HEK293T cells (Fig. 3A). Data filtering was performed as previously^10^ (see “Methods” for details). Using SciPhy, we conducted a phylodynamic analysis to estimate the lineage tree, the editing dynamics of the integrated barcodes, or ’DNA Tapes’, and the growth dynamics of the sampled cell population (see “Methods” for details).

We estimated SciPhy’s editing model parameters for this dataset, namely an editing rate for each DNA Tape and the overall insertion probabilities. Our estimates show substantial variation in the insertion probabilities (Fig. 3B). Notably, the ’CAT’ insert had the highest probability (16%), whereas ’GCG’ had the lowest (≤1%). Although this variation—potentially due to differential pegRNA expression or hexamer efficiencies—was documented in the initial publication^10^, the UPGMA tree-building method used there did not take it into account. Under the SciPhy model, we infer that when an insertion occurs, there is approximately a 50% chance that it will be one of the four most prevalent inserts (CAT, CCG, GCC, ATA). Consequently, these particular edits have a high chance of being acquired concurrently in otherwise independent lineages. Unlike UPGMA, SciPhy can account for this process by modeling these insertion probabilities, potentially avoiding the reconstruction of incorrect lineage relationships.

We further estimate an editing rate, or clock rate, for each DNA Tape, which denotes the rate at which edits (inserts) are introduced at target sites within that tape (Fig. 3C). Most DNA Tapes show clock rates of around 0.15 d^–1^, corresponding to an average of four edits per tape over the entire experiment, a value that aligns with our dataset observations (see Supplementary Fig. 5).

Interestingly, all four truncated DNA Tapes, i.e., tapes with fewer than five target sites (see “Methods” for details), showed elevated clock rates (Fig. 3C, first four tapes). Among these, the most truncated tape, ‘TTCACGTA’—shortened to just two sites (hereafter denoted as a 2x tape)—had the highest rate. The observed pattern of editing rate ordering by tape length is unexpected under a null model where all tapes have similar editing rates independent of their length. We therefore asked whether early edits occur at a different rate from late edits. We compiled a dataset in which all tapes were shortened to a length of two and compared the estimated editing rates with those of the original data encompassing all sites (Supplementary Fig. 6). Interestingly, the 2× tape ‘TTCACGTA’ is edited the fastest in both analyses, indicating that its elevated editing rate is not due to the first two edits occurring faster across tapes of any length. Given that we only have a single instance of this 2× tape, it is difficult to draw definitive conclusions, but one possible explanation is that it was integrated into a genomic location that facilitates a high editing rate.

We then compared the 4× and the 5× tapes. In the analysis using all sites, the three highest editing rates among the 4× and 5× tapes were all associated with 4× tapes. This result is surprising and statistically significant under the assumption of equal rate (*p*(*k* = 3) = 0.005, see “Methods” for test details). However, in the analysis using only two sites, this order was lost, with only one 4x tape among the three tapes with the fastest editing rates. This result was not statistically significant under the assumption of equal rates (*p*(*k* = 1) = 0.5). Therefore, given the editing rate estimates under SciPhy, the difference in editing rate between 4× tapes and 5× tapes may be due to a difference in editing speed across the last 2–3 sites, for instance, slower editing at the fifth site of the 5× tapes. To further explore this scenario, we simulated data in which the fifth site was edited at 20% of the original rate. In this scenario, but not under equal edit rates, we observed two 4× tapes being among the three tapes with the highest inferred editing rates (see Supplementary Fig. 7), supporting our earlier statistical analysis. However, as our HEK293T analysis is based on a single experimental replicate, additional replicates would be required to draw definitive conclusions on these relative edit rates.

Along with estimating the editing model, we studied the growth dynamics of the HEK293T cell population by fitting a birth-death-sampling model to the set of sequences. These sequences are a subsample of 1000 cells from a population reported to consist of approximately $1.2*{10}^{6}$ cells^10^. To incorporate this knowledge, we initially fixed the sampling proportion to $8*{10}^{-4}$, assuming constant population growth throughout the experiment. This model estimated a cell population growth rate between 0.42 and 0.47 per day (95% highest posterior density (HPD) interval, see Supplementary Fig. 16) “SciPhy + Fixed sampling”), corresponding to doubling times of 35–40 h. This growth rate, under an exponential population growth model, implies a population of $\approx {10}^{5}\pm {10}^{5}$ (mean ± *σ*) cells by the experiment’s end, which is an order of magnitude smaller than the reported $1.2*{10}^{6}$ cells. After confirming that this growth rate estimate was driven by the data in the tape alignments (see Supplementary Fig. 17), we hypothesized that this disagreement may stem from model misspecification. Therefore, we relaxed two of the main assumptions made in our model separately: first, the assumption of constant growth over the experimental period, and second, the assumption of an accurately known sampling proportion.

First, we ran the same analysis with piecewise constant birth and death rates, allowed to vary every 2 days over the timeline of the experiment. The estimated growth rates imply a pattern where the median growth rate is highest during the first 2 days of the experiment, with a median growth rate of 1.69 d^−1^ or doubling time of 9.6 h (95% HPD of growth rate [0.57, 2.76] d^−1^), and declines for 5 days after which it stabilizes around 0.3 d^−1^ until the end of the experiment (see Supplementary Fig. 15). Under this set of rates, the cell population grows to an expected size of $\approx 9*{10}^{5}$ cells, closer to the observed $1.2*{10}^{6}$ cells. However, the fast doubling time in the first days is unrealistic for HEK293T cell growth from a single cell. Furthermore, it should be noted that a constant growth rate over the entire experimental period is still not excluded under these results.

Second, we relaxed our assumption of accurate knowledge of the sampling proportion by jointly estimating it with the growth rate. This analysis inferred a growth rate between 0.49 and 0.58 per day, or a doubling time that ranges from 28 to 33 h (95% HPD interval, Fig. 3D). This doubling time is consistent with documented HEK293T doubling times^20^ and leads to an average population size between $2.5*{10}^{5}$ and $2.3*{10}^{6}$ (95% HPD) after 25 days, which agrees with the reported $1.2*{10}^{6}$ cells.

Interestingly, the inferred sampling proportion under the last model is approximately four to 16 times lower than initially assumed (95% HPD interval: $[5.2*{10}^{-5},2.2*{10}^{-4}]$, Fig. 3E). Under the birth-death model parameterized for this analysis, a lower-than-expected sampling proportion may stem from measurement errors in estimating the total cell population size—potentially caused by cell clumping during counting—or from non-uniform sampling, such as biases in cell selection, sequencing, or filtering. Given that the reported population size of $1.2*{10}^{6}$ cells was measured using both a hemocytometer and a cell counter, which should provide reliable counts, it is more plausible that biases in the sampling process led to this reduced estimate. In such cases, the inferred sampling proportion likely reflects an ‘effective’ sampling proportion, accounting for these underlying biases rather than the true biological sampling proportion. Finally, we performed sensitivity analyses using alternative prior assumptions for the editing model parameters and found that our results remained robust across these settings (Supplementary Fig. 21).

Taken together, the three analyses presented here suggest that a model of constant growth adequately describes the population dynamics under study.

### Comparison to state-of-the-art

**Fig. 4:**
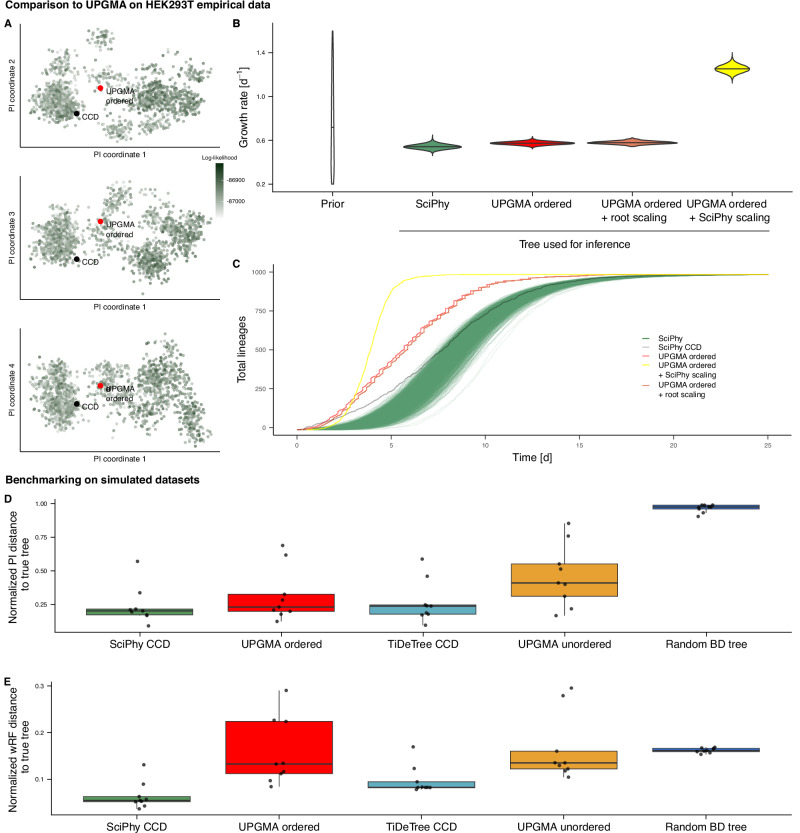
SciPhy produces distinct and more accurate estimates on real and simulated data, respectively. In panels **A**–**C**, we show estimates obtained using UPGMA and SciPhy using the HEK293T dataset as input. In **A**, we compare the SciPhy posterior tree set (green, darkness denotes the log-likelihood value), including a summary tree, the conditional clade distribution (CCD) tree (black), to the tree reconstructed with the order-aware UPGMA method (red). We do so by calculating the Phylogenetic Information (PI) distance that captures topological distances between all pairs of trees. We then use multidimensional scaling (principal coordinates analysis) to project these distances into 12 dimensions. Here we show the results of the first coordinate against coordinates 2–4 from top to bottom. The remaining pairwise coordinates are shown in Supplementary Fig. 18. In **B**, we compare growth rates jointly estimated with the SciPhy posterior tree set inferred under the constant birth-death-sampling model (green and the summary CCD in gray), order-aware UPGMA (red), order-aware UPGMA with scaled root height (coral), and order-aware UPGMA scaled using SciPhy (yellow). In **C**, we plot the total number of lineages (*y*-axis) through time for each day (*x*-axis) for the trees reconstructed by each of the methods. In **D** and **E**, we extend this comparison using simulated datasets (*n* = 9) and additionally compare SciPhy to counterpart methods that do not model ordered editing, i.e., TideTree (light blue), and standard, order-unaware UPGMA (‘UPGMA unordered’, orange). For all box plots, the center line represents the median distance (PI in (**D**) and weighted Robinson–Foulds (wRF) in (**E**)) to the true tree; bounds of the box represent the 25th and 75th percentiles; whiskers extend to the minima and maxima, excluding outliers; all individual distances are overlaid as points. Source data are provided as a Source Data file.

We compare SciPhy with existing methods for phylogenetic tree topology reconstruction on simulated and real data in Fig. 4. For the comparison on simulated data, we reuse a subset of nine datasets from the validation study (see “Methods” for details). We contrast SciPhy with current approaches that use order-aware UPGMA clustering. We also compare against methods that do not use the ordering information from the DNA Typewriter data, namely TiDeTree and UPGMA clustering based on an order-unaware distance matrix. As a baseline, we included random trees simulated under a birth–death model with the same number of tips.

We find that SciPhy’s tree point estimate based on the conditional clade distribution (CCD) achieved the lowest median Phylogenetic Information (PI) distance to the ground truth tree, indicating the highest reconstruction accuracy across methods (Fig. 4D). Notably, TiDeTree performed comparatively well despite not having access to the true order of edit acquisition. However, we expect the performance gap between any method only modeling unordered edits and SciPhy to widen in scenarios where the edit distribution is more skewed—i.e., where some edits are much more frequent than others—since having access to the true order of edits becomes increasingly informative in such settings.

To evaluate the combined performance of tree topology and branch length estimation, we compared the estimated trees in terms of the weighted Robinson-Foulds distance (wRF; Fig. 4E). Here, SciPhy clearly outperforms all other methods. This advantage arises from SciPhy’s explicit model of sequential edit acquisition within a tape. In contrast, both TiDeTree and unordered UPGMA treat all sites as independent and are thus misspecified. Ordered UPGMA incorporates site ordering in the distance matrix calculation (i.e., a higher similarity comparing sites within tape sites is only achieved if the previous sites are identical). However, ordered UPGMA clustering only uses pairwise distances and does not use the known ancestral state of each tape, nor does it use the knowledge of the editing process to inform branch length inference as SciPhy does (see Supplementary Figs. 3 and 4 for more assessment of the accuracy under varying parameter regimes).

To showcase how the differences highlighted above on simulated data may translate to empirical analyses, we repeated the comparison between SciPhy and the order-aware UPGMA algorithm using the HEK293T dataset as input to both methods. Again, to investigate whether they recover similar ancestral relationships between cells, we used the Phylogenetic Information metric to compute pairwise distances between sets of SciPhy posterior trees and UPGMA, visualized in four coordinates (Fig. 4A and see Supplementary Fig. 10). Within this metric space, the UPGMA tree falls separate from the bulk of our inferred set of posterior tree topologies in half of the pairwise coordinates (coordinates 1–2, 1–3 and 1–4, see Supplementary Fig. 10 for all pairwise coordinates), indicating that both methods capture similar overarching topological features, but that SciPhy recovers distinct cellular relationships looking at a finer resolution. We find similar patterns of segregation between the SciPhy posterior and UPGMA trees using different distance metrics. Using the Robinson-Foulds distance, the UPGMA topology shows an overlap with SciPhy posterior tree estimates in all but the first two pairwise coordinates (Supplementary Fig. 8). Consistently, the Clustering Information distance showed separation between UPGMA and SciPhy trees in two out of six pairwise coordinates (coordinates 1–2, 1–3 in Supplementary Fig. 9).

Next, we also evaluated SciPhy against the order-aware UPGMA with respect to branch length inference for the HEK293T dataset. UPGMA produces trees with relative branch lengths that are not anchored to an absolute time scale. In contrast, SciPhy calibrates branch lengths to absolute time, using the experiment’s duration as a temporal reference. For fair comparison, we adapted the UPGMA tree to include temporal information by scaling it in three different ways. First, we scale its root (the most recent common ancestor (MRCA) of all cells, representing the timing of the first division event) to 25 days to match the experiment duration (labeled “UPGMA ordered”). However, it is likely that this first division event happened after the start of the experiment. Therefore, we also scaled the UPGMA root to the median root height of SciPhy trees (labeled “UPGMA ordered + root scaling”). Finally, we also estimate branch lengths using SciPhy on the fixed UPGMA topology (labeled “UPGMA ordered + SciPhy scaling”).

Adopting the same approach as before to visualize trees in pairwise dimensions but using the weighted Robinson-Foulds distance, accounting for branch lengths, we observe that UPGMA trees diverge from trees in our posterior set in all pairwise coordinates (see Supplementary Fig. 12). Given the topological similarities previously noted, this implies that UPGMA predicts branch length patterns distinct from those in trees from SciPhy’s posterior set.

To further illustrate these branch length differences, we display the total number of lineages through time (LTT) for each reconstructed phylogeny (Fig. 4B). Notably, SciPhy posterior trees display a slower increase in the number of lineages compared to UPGMA. For example, 500 lineages are reached after 5.5 days according to the UPGMA tree (5.7 days in the scaled UPGMA), but only after a median of 8.8 days (95% HPD interval of [8.0, 9.7]) under SciPhy. Similarly, in the UPGMA tree, the final number of 1000 lineages is reached 1.1 days earlier (median, 95% HPD interval [0.31, 2.1]) compared to trees estimated by SciPhy. As a result, there is no overlap in LTT between the SciPhy tree posterior and the UPGMA tree for most of the experimental duration.

Branch lengths are the key signal informing population dynamics in phylodynamic analyses. Given the divergence in estimated branch lengths between UPGMA and SciPhy, we investigated the impact on growth rate estimates. We compared growth rate estimates based on four input tree sets: (1) the posterior distribution of trees (“SciPhy”), (2) the UPGMA tree scaled to the full experimental duration (“UPGMA ordered”), (3) the UPGMA tree scaled to the median root height of SciPhy trees (“UPGMA ordered + root scaling”), and (4) the UPGMA tree with SciPhy-estimated branch lengths (“UPGMA ordered + SciPhy scaling”) (Fig. 4C). The median growth rate inferred on fixed UPGMA trees (95% HPD interval of [0.54, 0.60] (2), [0.55, 0.60] (3) and [1.19, 1.32] (4)) is higher than the one inferred under SciPhy. Notably, the growth rate inferred on the UPGMA topology with branch lengths scaled with SciPhy is the highest, leading to doubling times between 12.5 h and 14.0 h, which excludes reported values for this cell line.

Overall, these results highlight that, consistent with the differences underlined in simulated data, empirical trees reconstructed using SciPhy and UPGMA for the HEK293T dataset differ in topology, although the relative impact of these disparities is challenging to quantify in the absence of a ground truth for this empirical dataset. There are notable differences in the branching times, which can lead to qualitative differences in the estimated growth rates.

### Estimating growth dynamics during gastruloid development

**Fig. 5:**
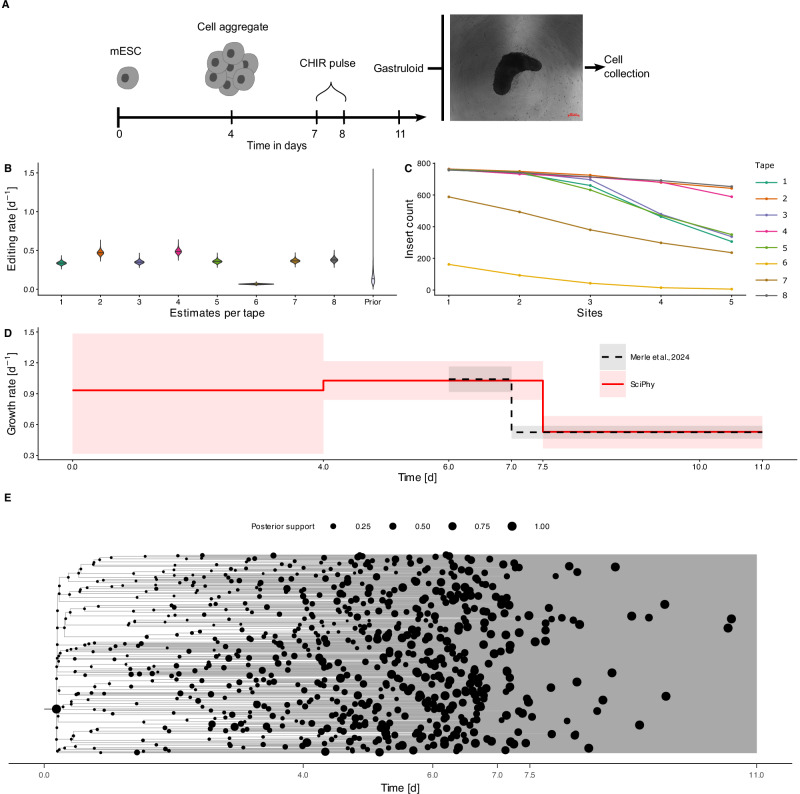
SciPhy estimates a slowdown in growth rate upon CHIR stimulation. **A** We apply SciPhy to a dataset of 780 tape sequences collected from a monoclonal expansion experiment of gastruloid growth (*n* = 1). After 4 days of growth, cells form an aggregate in which they continue growing until day 7, when a CHIR pulse is applied to trigger elongation into a gastruloid structure. At day 11, the cells are collected for sequencing. **B** We show the insert count for each tape across all sites. In **C**, we display the estimated posterior distribution and median (center line) edit rate per day for each tape. In **D**, we show the estimated time-varying growth rate of the cell population (median and 95% HPD) against the growth rates (mean $\pm \sigma$) measured in ref. ^21^, binned to match our time intervals (see “Methods”). **E** Is the inferred CCD lineage tree inferred under SciPhy for this dataset. Source data are provided as a Source Data file.

We analyzed a dataset where DNA Typewriter was employed to record the process of gastruloid formation. The experiment began with a single mouse embryonic stem cell (mESC), which was cultured as a single colony on top of mouse embryonic fibroblasts over 4 days, after which it was further expanded as a spherical 3D culture for 3 days. Then, Chiron (CHIR) treatment was applied for 1 day, initiating symmetry breaking and elongation in the gastruloid. Finally, the cells grew for 4 more days before being collected for sequencing (Fig. 5A). Our analysis focuses on 780 cells, each of which contained the same set of the eight most frequent DNA Tapes.

Initially, we visualized the dataset by displaying the number of inserted sequences per site for each tape (Fig. 5B). The data reveal differential editing across tapes. For instance, tapes 2, 4, and 8 exhibit high levels of editing across all sites, whereas the other tapes show a stronger decline in the number of inserts at the later sites. Notably, tape 6 is sparsely edited, with fewer than 200 cells being edited at the first site.

In addition to the overall number of inserts, the number of uniquely edited tape sequences provides more intuition about the information content in the different tapes. Thus, we also examined the number of unique sequences for each tape (Supplementary Fig. 19). Interestingly, the most heavily edited tapes (2, 4, 8) show the smallest number of unique sequences, indicating that their editing saturated early in development when fewer cells were present. Conversely, a higher number of unique sequences were found in tapes 1, 3, 5, and 7, despite being less edited. This could be due to editing being slower and thus lasting longer, thereby marking more cells. Despite being the least edited tape, tape 6 shows a number of unique sequences comparable to the heavily edited tapes.

We apply SciPhy to estimate the editing model parameters, the time-scaled phylogeny, and a dynamic growth rate over time. We find that the estimated clock rates vary among the different tapes (Fig. 5C). Notably, tapes 2 and 4, which were suspected to be edited early based on the insert count analysis, also exhibit elevated editing rates based on our phylogenetic analysis. Additionally, the editing rate for tape 6 is notably lower compared to other tapes, consistent with its lower insert count. We further estimated the insertion probabilities for all inserts and found a highly unbalanced pattern (Supplementary Fig. 20). Specifically, there is approximately a 50% probability of inserting the sequences “CAC” or “CCG”. This suggests that independent insertions of the same edit in the same tape are likely, which SciPhy accounts for.

To estimate the growth rate of the gastruloid over time, we use a birth-death skyline model with varying birth and death rates. One advantage of the mechanistic nature of this model is that it allows us to directly set the rate changes to specific time points aligned with experimental steps. For instance, we allow the rates to change after day 4, when the cells start to grow within an aggregate. Additionally, we allow for another change in rates after CHIR treatment, at day 7.5, leading to two change points in total (Fig. 5D). During the expansion from a single-cell to aggregate, we estimate a median growth rate of approximately 0.9 d^−1^ (95% HPD of [0.34, 1.50]), which corresponds to a doubling time of 18.5 hours. Between days 4 and 7.5, the median growth rate is estimated to be slightly elevated to 1 d^−1^ (95% HPD of [0.85, 1.2]). After the CHIR pulse, we infer a significantly lower growth rate compared to the previous interval, with a median of 0.5 d^−1^ (95% HPD of [0.36, 0.67]). A decrease in growth rate after day 8 is also recovered when allowing rate changes at days 7 and 8 (before and after CHIR pulse, see Supplementary Fig. 18 “3 change times”), suggesting that this observation is robust to different timeline setups. This slowing population growth can be visually appreciated from the time-scaled phylogeny (Fig. 5E), where the number of new branching points (cell division events) decreases after day 8. We further performed various sensitivity analyses and found that our results remained robust across all settings (Supplementary Fig. 22). As empirical validation of this pattern, we compare our estimates to previously reported growth dynamics of gastruloids recovered through imaging approaches^21^ (Fig. 5D). The magnitude of the decrease in growth rate is in close agreement across both methods. However, our estimates suggest the decrease may have occurred 12 (Fig. 5D) to 24 h (Supplementary Fig. 18) “3 change times”) later than reported by Merle et al.^21^. One possible reason for this discrepancy is our assumption of a constant editing rate throughout the experiment. If the rate of DNA Tape editing declines over time, as observed with another sequential prime editor^22^, some cell divisions may be erroneously placed at later time points, resulting in the delayed decline in growth rate. Another factor could be differences in experimental protocols, as our dataset derives from monoclonal expansion, whereas Merle et al.’s was generated from an initial aggregate of several hundred cells. Despite the uncertainty regarding the exact timing of the growth rate decline, both datasets contain a signal for such a decline, suggesting a conserved response to CHIR signaling, independent of growth protocol.

In summary, our analysis reveals a pattern of slowed cell divisions following CHIR treatment in this gastruloid, confirming previous observations^21^. Importantly, this example showcases that lineage tracing data alone was able to encode a signal for time-varying growth dynamics previously obtained manually through a combination of imaging and segmentation.

## Discussion

In this study, we introduced an editing model, an edit sequence simulator, and a likelihood calculation tailored for lineage recordings based on sequential genome editing. Packaged as ’SciPhy’ (**S**equential **c**as9 **i**nsertion based **Phy**logenetics), our model is integrated into the BEAST 2 Bayesian phylogenetics framework. This integration empowers SciPhy users to specify complex editing dynamics, for example, varying editing rates across DNA Tapes as demonstrated in this work, or along tree branches (using, e.g., relaxed clock models^23,24^), which we did not explore. Additionally, it supports more nuanced models of cell development, allowing for cell-type dependent division and differentiation rates using multi-type models^25,26^ or time-varying rates^27,28^. The BEAST 2 platform is increasingly attracting developers interested in single-cell analyses^14,29–31^, and we foresee that support in this area will continue to grow in the future.

Our evaluations of SciPhy on experimental datasets demonstrate its ability to capture complex editing dynamics, such as the presence of preferential insertions and editing rate variation between tapes, patterns that are prevalent in both sequential lineage datasets we analyzed and those from other lineage tracing technologies^2,14,32,33^. As a mechanistic model, SciPhy can directly account for these factors and even use the signal from tapes being edited at different rates to inform branch length estimation. This ability to estimate branch lengths, and from them, cell population dynamics, is consistent with a growing body of related work assessing properties of sequential editing data^34,35^.

We compared SciPhy to existing phylogenetic methods on both simulated and real data. In our simulated benchmark, we showed that SciPhy outperforms all existing methods in terms of tree reconstruction accuracy and branch length estimation. We further compared SciPhy to ordered UPGMA clustering, which was used in both previously published analyses, on real data. UPGMA does not account for preferential insertions nor different edit rates for different tapes. We find that some—but not all—aspects of the tree topology are captured in both SciPhy posterior trees and the UPGMA tree. Notably, the branch lengths differ significantly, which we showed impacts the downstream inference of the cell population growth rate and is likely to impact other parameters one may wish to estimate from cell phylogenies in the future, such as cell-type differentiation rates.

For both datasets analyzed in our study, we used phylodynamic models to estimate cell population growth based on SciPhy’s posterior distribution of timed trees. In HEK293T cells, this approach showed that sequential lineage tracing data alone contain a signal for the growth rate characteristic of this cell line. When analyzing murine gastruloid development, we estimated dynamic growth rates over experimentally informed time intervals and observed a significant reduction in cell growth after CHIR stimulation, consistent with previously reported dynamics^21^. This is also aligned with recent research aiming to resolve mechanisms of axis elongation during gastruloid development^36^, suggesting that elongation occurs not through increased, localized cell division, but rather through active cell movement and differential adhesion. In both analyses, SciPhy allowed us to extract relevant, previously inaccessible insights from the lineage tracing data.

In our analysis of cell culture growth, we observed that the inferred editing rates for the 4-site tapes were consistently higher than those for the 5-site tapes. We used a statistical test to show that this pattern is unlikely to arise under a null model of equal rates across all tapes. However, a limitation of our approach is that all inferences were performed using SciPhy, which assumes equal editing rates across sites. We present simulations that provide support for SciPhy estimating an average edit rate across sites for each tape, essentially allowing us to constrain the space of within-tape rate variation to scenarios consistent with the estimated per-tape average. Nevertheless, a model that explicitly allows for site-specific editing rates would enable more direct testing of such hypotheses and remains an important direction for future work.

While SciPhy offers promising avenues, it has some limitations. Tree space grows exponentially with the number of cells, and current state-of-the-art Bayesian phylogenetic inference methods may not converge for trees with more than around 1000 tips. Bayesian phylogenetic inference requires repeatedly evaluating the likelihood to reach convergence. Thus, despite the likelihood calculation scaling linearly with the number of tips, further speed-ups of the likelihood calculation can bring large benefits. We have achieved considerable improvements on the runtime of individual computations of the likelihood with caching and multi-thread parallelization (and provide estimates of the resulting combined, overall runtime for convergence of typical analyses in Supplementary Figs. 9 and 10). However, the empirical data analysis on 1000 cells still required ~15 days, and would benefit from further reduction in runtime. For future improvements, we aim to accelerate the likelihood calculation using software optimizations or approximations, and expedite convergence on large datasets by confining tree space and adapting recent advancements based on mutation-annotated trees^37^ that have been demonstrated to scale to 100,000 tip trees. Improvements to other components of Bayesian inference, such as, e.g., MCMC sampling methods, may also provide additional runtime reduction^38,39^ but their applicability beyond continuous/real parameter contexts, such as the one encountered in phylogenetic inference, remains an open research problem.

Another limitation shared by all CRISPR-based barcoding technologies is that resulting lineage tracing datasets are typically lossy as a result of the sparseness of single-cell RNA-seq and/or silencing of the regulatory elements that drive transcription of recording substrates (e.g., DNA Tapes). Jointly, these mechanisms result in the partial recovery of DNA Tapes per cell. For example, in the HEK293T cell culture dataset used in our study, none of the tapes were recovered for all sequenced cells, and there was variability in the rate of recovery of individual tapes. The relative contribution of either mechanism to this loss is unknown, and for ordered lineage tracing data, state-of-the-art lineage reconstruction approaches have thus far neglected cells with incomplete DNA Tapes. In our simulations, we found that trees reconstructed from incomplete alignments of DNA Tapes are less accurate, but that parameters inferred jointly with those trees remain overall unbiased (see Supplementary Fig. 25 for the editing and growth rates). However, ignoring such sources of loss may still bias tree or population parameter inference by violating a common assumption of birth-death models regarding uniform sampling of the population of interest, e.g., if DNA Tapes are simultaneously lost for groups of related cells. Related work for single-cell diploid data and CRISPR-based unordered data has shown that mechanistic models of error sources improve features of inferred lineage trees^29,40^, especially relating to branch lengths. Follow-up work should integrate a dropout model accommodating tape absence such that data loss and the resulting bias are avoided, and thoroughly investigate more complex patterns of loss. For the datasets analyzed in this study, sequencing error is unlikely to be a major issue, as the tapes are recovered using UMIs (any retained error would thus need to arise multiple times independently). For datasets relying on other sequencing techniques, extending SciPhy with explicit models of such error may, however, become relevant follow-up work.

Many challenges remain in reconstructing accurate lineage trees, including refining experimental designs to improve signal quality in lineage tracing data. Future studies should systematically compare sequential genome editing with previous unordered methods, assessing both tree topology and time estimates. Such comparisons will be essential to guide experimental efforts.

Lineage recorders based on sequential genome editing represent a notable advancement as they encode not only a number of edits but also the order in which they occur. With SciPhy, we present a model that directly captures and uses this detailed information, which we expect will lead to improved tree reconstruction compared to commonly used clustering tools. We see great promise that sequential lineage recorders capable of co-tracking cellular signals^22^, will be developed in the future. In parallel, SciPhy can be expanded to inform about the timing of such recorded signaling events in addition to the single-cell trees, leading to developmental insight in ever-greater detail.

## Methods

### Editing model for barcodes with ordered inserts

We present a model that captures the editing dynamics underlying the generation of sequential lineage tracing data using DNA recording systems such as the ones recently developed by refs. ^9,10^. In these systems, cells carry stably integrated barcodes or “tapes”, which are contiguous arrays of CRISPR-Cas9 target sites. Within these tapes, target sites are sequentially made amenable to prime editing, resulting in the ordered accumulation of inserts. We consider an experimental setup starting with a single cell carrying several of these unedited tapes. As this cell and its offspring undergo proliferation, their tapes experience editing. At the end of the experiment, single cells are sampled and their tapes sequenced. Aligning sequenced tapes to their corresponding, unaltered tape reveals genetic modifications accumulated during the experiment. In the following, we detail the notations and assumptions we use to model this process.

Consider the following setup. Each cell carries *k* tapes that independently undergo editing. Each unedited tape is an array of N target sites, numbered from 1 to *N*. Each site can acquire a single insertion or edit, with *M* different inserts available.

Model definitions:

1. Initially, only a tape’s first site is susceptible to editing. Subsequent sites are edited sequentially: editing at a site ${i}_{1 < i\le N}$ is only possible if its preceding site $i-1$ is already edited.
2. Editing is a two-step process:
  - Step 1: The Cas9 prime editor introduces cuts at the target sites in a time-dependent manner at a rate *r*, called the editing rate or clock rate. This means editing follows a bounded Poisson process, which stops once *N* edits have occurred.
  - Step 2: pegRNA-mediated insertions following cut events are instantaneous. The set Γ of *M* possible insertions is predefined and denoted as:
  1 $$\Gamma=\{{\gamma }_{1},{\gamma }_{2},...,{\gamma }_{{M}}\}$$
  Given a cut event, the probability of adding insert ${\gamma }_{{i}}$ is ${f}_{{i}}$ (with $\eta :=({f}_{1},{f}_{2},...,{f}_{{M}})$), allowing for some insertions to have higher probabilities than others. We additionally assume that insertions operate independently—an insertion at a previous site does not influence the insertion probability at the currently active site.
3. Editing of any target site is irreversible.

From the model definition, it follows that the number of edits introduced in each independent tape is Poisson distributed. However, the distribution of the total number of edits per cell is not guaranteed to show features of a classic, unimodal Poisson distribution, because it is also influenced by the shared ancestry of the sampled cell population and its growth patterns (see Supplementary Figs. 13 and 14).

Formally, for an array of length *N*, this model of editing is a continuous-time Markov chain on the state space Ω of all possible insertions and parameters $r,\eta$. These states are all possible finite sequences of length $n\le N$, or *n*-tuples of ordered insertions, that we denote as:

2 $$({s}_{{i}})_{{{{\rm{i}}}}\le n},{s}_{{i}}\in \varGamma$$

where subscript *i* denotes the insertion site in the tape, i.e., its order. Note that the empty tuple *∅* represents the unedited state or starting state for all *k* tapes sampled per cell, and the fully edited tape is an absorbing state of the Markov chain.

Let ${Y}_{t}$ be the state of an array at time *t* after the start of the experiment. Given an array state ${Y}_{t}=a$ and its state after a time interval $\Delta t$, ${Y}_{t+\Delta t}=b$, we denote the tuple of inserts added to *a* to result in *b* as *c*. Specifically, *c* represents the elements in *b* that are not in *a*, which can be expressed as $c=b\backslash a$. The size of this tuple, denoted as ${|c|}$, corresponds to the number of inserts introduced between states *a* and *b*.

### Transition probabilities

We denote the transition probability between states a and b under the model introduced above as ${P}_{{a}{,}{b}}(\Delta t;\eta,r)=P({Y}_{t+\Delta t}=b;{Y}_{{t}}=a,\eta,r)$. In the following, we derive this transition probability. In step 1, we calculate the probability that a number of $|{{{\rm{c}}}}|$ new edits have been inserted. In step 2, we calculate the probability that those edits correspond exactly to the tuple c.

**Step 1** To derive the probability of introducing ${|c|}$ edits, we distinguish two cases. In case 1, the number of new inserts ${|c|}$ does not lead to saturation in the array ($|a|+|c|=|b| < N$). Then, the probability of ${|c|}$ editing events happening at rate $r$, in time $\Delta t$ is given by the Poisson distribution (by model definition 2a):

3 $$P(|c|=j;r,\Delta t,|b| < N)=\frac{{(r\Delta t)} ^{ \, j}}{j!}{e}^{-(r\Delta t)}$$

In case 2, introducing $|c|$ inserts leads to saturation of the array ($|a|+|c|=|b|=N$), i.e., reaching position *N* in the tape. We calculate the probability of this event by targeting its complement, namely the probability that less than $|c|$ inserts were introduced (again, by model definition 2a):

4 $$P(|c|	=j;r,\Delta t,|b|=N)=1-P(|c| < j;r,\Delta t,|b| < N) \\ 	=1-{\sum }_{k=0}^{j-1}P(|c|=k;r,\Delta t,|b| < N)$$

Combining both cases, the probability of introducing ${|c|}$ edits in an array of length N given an editing rate $r$ is:

5 $$P\left(\left|c\right|=j;r,\Delta t\right)=\left\{\begin{array}{cc}\frac{{(r\Delta t)}^{j}}{{{{\rm{j}}}}!}{e}^{-\left(r\Delta t\right)} & {{\rm{if}}}\left|b\right| < N.\\ 1-{\sum }_{k=0}^{j-1}\frac{{(r\Delta t)}^{k}}{k!}{e}^{-\left(r\Delta t\right)} & {{\rm{otherwise.}}}\end{array}\right.$$

**Step 2** Let us assume that the ${|c|}$ insertions that occurred from a to b are exactly the tuple $c=({s}_{i})_{i=|a|+1,\ldots,|b|},{s}_{{i}}\in \varGamma$. Conditional on observing exactly $|c|$ inserts, the probability of realizing the specific tuple *c* equals the product over the insertion probabilities ${f}_{l}$ for each element of *c*, (by independence, see model definition 2b):

6 $$P\left(c;\left|c\right|=j,\eta \right)={\prod }_{k=\left|a\right|+1}^{\left|a\right|+j}P\left({s}_{{k}}\right)={\prod }_{k=\left|a\right|+1}^{\left|a\right|+j}{\prod }_{l=1}^{M}{{f}_{{l}}}^{{{{\bf{1}}}}\left({\gamma }_{{{{\rm{l}}}}},{s}_{{k}}\right)}\,$$

Note that the indicator function ${{{\bf{1}}}}({\gamma }_{l},{s}_{k})$ serves as a one-hot selector, keeping the insertion probability ${f}_{l}$ only when the edit ${\gamma }_{l}$ matches the observed symbol ${s}_{k}$ in the tuple *c*. This construction collapses the inner product to the single insertion probability corresponding to the realized edit at position $k$.

**Fig. 6:**
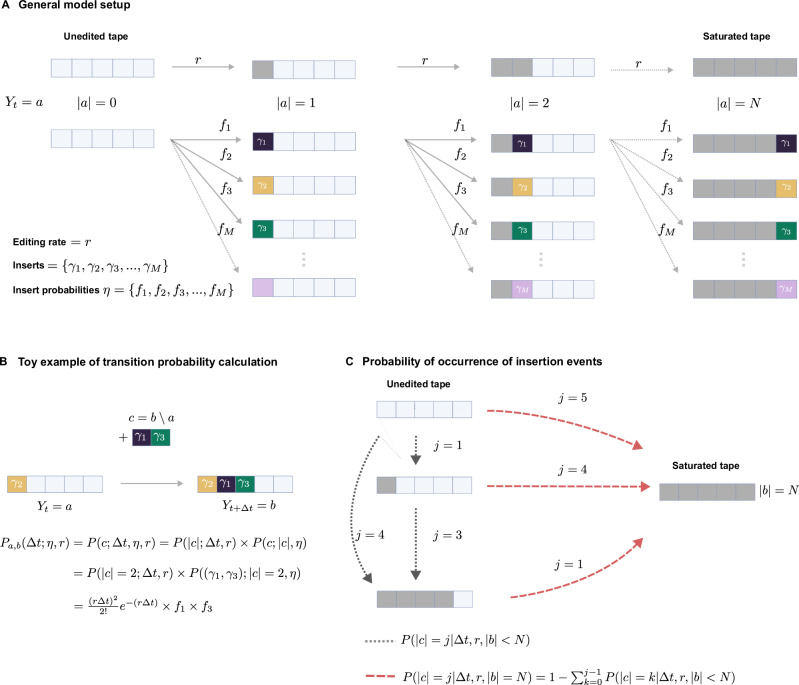
General description of model components used to calculate transition probabilities in SciPhy. An overview of SciPhy’s editing model for a tape with *N* = 5 target sites and its parameters is shown in (**A**). Target sites within tapes (rectangles in the tape) acquire inserts (colored boxes, inserts ${\gamma }_{1}$ to ${\gamma }_{M}$) with probability ${f}_{1}$ to ${f}_{M}$ following Cas9-mediated nicks at rate $r$. Upon editing, these sites are inactivated (gray boxes). **B** is a toy example for the calculation of the transition probability for a tape acquiring 2 edits (${\gamma }_{1}$ and ${\gamma }_{3}$). In **C**, we showcase different scenarios for the calculation of the probability of a given number of inserts to be added into a tape, distinguishing between cases where a tape gets saturated with inserts (red arrows pointing to the right) or not (gray arrows pointing down).

**Step 3** Combining the steps above, the transition probability between any two states a and b in time interval $\Delta t$ given model parameters $r$ and $\eta$, is obtained as follows (and as described in Fig. 6):

7 $${P}_{{a}{,}{b}}\left(\Delta t;\eta,r\right)	=P\left({Y}_{t+\Delta t}=b;{Y}_{{t}}=a,\eta,r\right)=P\left(c;\Delta t,\eta,r\right)\\ 	=P\left(\left|c\right|;\Delta t,r\right)\times P\left(c;\left|c\right|,\eta \right)$$

where $P(|c|;\Delta t,r)$ was derived in Step 1 and $P(c;|c|,\eta )$ in Step 2.

### Likelihood calculation

The editing model defined above can be used to calculate the likelihood of the model parameters $(r,\eta )$ and the cell phylogeny $T$ given the tape data $D$ at the tips, ${{\mathrm{Lik}}}(r,\eta,{{\mathrm{T}}};D)=P(D;r,\eta,{{\mathrm{T}}})$. Let ${{{\rm{T}}}}$ be a tree with $n$ tips and $n-1$ internal nodes. The oldest of these nodes is the root node, being the most recent common ancestor of all sampled cells. Additionally, we introduce a stem branch preceding the root, connecting the root node to the start of the experiment, or origin node. We number the leaf nodes from $1,...,n$ arbitrarily, and the internal nodes from $n+1,...,2n$ with increasing distance from the present. Each cell may have $k$ tapes. Since we assume that tapes are edited independently from each other, we calculate the probability density of tape $i$ given the tree and $r,\eta$ separately for $i=1,...,k$. The likelihood is then the product of the $k$ probability densities. In what follows, we thus consider one tape.

We first obtain the sets of possible tape states at each internal node, given the data at the tip nodes. The likelihood of the tree is then calculated by summing over all possible state transitions between the nodes. This summation is performed through dynamic programming, going from the leaf nodes toward the origin of the tree; the employed particular algorithm is Felsenstein’s pruning algorithm^41^.

Hence, we first determine all possible states at internal nodes. We restate that editing is irreversible and ordered (model definitions 1, 3). Therefore, for any sequence at a leaf node, ancestors can only harbor either the same or fewer edits. For any leaf node $m$, let ${v}^{m}$ be its tape with elements ${v}^{{m}}=({s}_{{i}}^{{m}})_{|{v}^{{m}}|}$ where $|{v}^{{m}}|$ is the number of edits in ${v}^{m}$ as defined in the editing model section. The set of its possible ancestor tape states ${A}_{m}$ is the set of all subsets of edits from ${v}^{m}$, akin to a power set on ordered tuples:

8 $${A}_{{m}}=\{{{\varnothing }},({s}_{1}),({s}_{1},{s}_{2}),..,({s}_{1},{s}_{2},..,{s}_{|{v}^{{m}}|})\}.$$

For an internal node $i$ of tree $T$, it follows that the set of possible states ${A}_{i}$ is the intersection of possible states of its two children nodes $k$ and $j$, ${A}_{k}$, and ${A}_{j}$ (note that for the leaves, we initialize with all possible ancestor states). This is a result of editing being irreversible (model definition 3): an edit present in one child node but not the other cannot have been present in their parent node. Conversely, an edit present in both children nodes can either have been present in the parent node or have been acquired by both children independently; therefore it should be included in the set of possible parent states. Consequently, the possible state set for node $i$, ${A}_{i}$ is obtained as the intersection of the possible states of its children’s $k,j$, ${A}_{k}$, and ${A}_{j}$, recursively up the tree:

9 $${A}_{{i}}={A}_{{k}}\cap {A}_{{j}}$$

noting that the leaves are initialized with all possible ancestor states.

Given the possible states at the internal nodes, we calculate the probability density ${{{\rm{P}}}}({{{\rm{D}}}}|{{{\rm{T}}}},{{{\rm{r}}}},{{{\rm{\eta }}}},{{{{\rm{A}}}}}_{2{{{\rm{n}}}}})$ of the tape data given the tree and model parameters following notations in ref. ^14^. To match model definition 1, we assume the unedited state at the origin node ${A}_{{2}{n}}=\{{{\varnothing }}\left)\right.\}$.

10 $$\begin{array}{l}{{\mathrm{Lik}}}(T,\eta |D)=P(D|T,\eta,{A}_{{2}{n}})\end{array}$$

The transition probabilities from every parent node ${\pi }_{i}$ to its child node $i$ along their connecting branch with length ${\tau }_{i}$ are calculated using Eq. 3. Remember that ${A}_{i}$ is the set of all possible states at node $i$. Any specific state within ${A}_{i}$ is denoted as ${a}_{i}$. To account for all possible transitions, we sum the probabilities over all possible states at each internal node of the tree.

11 $$={\sum }_{{a}_{2n-1}\in {A}_{2n-1}}\ldots {\sum }_{{a}_{n+1}\in {A}_{n+1}}{\prod }_{i=1}^{2n-1}{P}_{{{a}}_{{{\pi }}_{{i}}}{,}{{a}}_{{i}}}\left({\tau }_{{i}},\eta \right)$$

Using Felsenstein’s pruning algorithm, we ‘move the summations down the tree as far as possible’^13^), meaning we perform the summations recursively up the tree, saving computations:

12 $$={\prod }_{i=1}^{2n-1}{\sum }_{{a}_{2n-1}\in {A}_{2n-1}}{P}_{{{a}}_{{{\pi }}_{{i}}}{,}{{a}}_{{i}}}^{{1}{(}{{\pi }}_{{i}}{,}{{a}}_{{2}{n}{-}{1}}{)}}({\tau }_{{i}}{,}\eta )\times \ldots \times {\sum }_{{a}_{n+1}\in {A}_{n+1}}{P}_{{{a}}_{{{\pi }}_{{i}}}{,}{{a}}_{{i}}}^{{1}{(}{{\pi }}_{{i}}{,}{{a}}_{{n}{+}{1}}{)}}({\tau }_{{i}}{,}\eta )$$

As stated above, this calculation of $P(D|T,r,\eta,{A}_{2n})$ is performed for each tape. Multiplying the resulting probability densities for each tape leads to the targeted likelihood function.

For a set of $k$ tapes, where every tape is of length *N* on a tree with $n$ tips, we perform *n* − 1 operations to calculate the set of ancestral states at every internal node. Performing the intersection at an internal node scales with the size of the set, *N*. Hence, computing the ancestral states scales in O(*knN*).

Additionally, for each of the *k* tapes, we calculate the transition probabilities at every one of the n-1 internal nodes. At each node, we consider a maximum of (*N* + 1)×(*N* + 1) potential state transitions. Hence, our likelihood computations without optimization scale in O(*kn*${{{{\rm{N}}}}}^{2}$) (In current technologies, *N* is relatively small, $N\approx 5$). Note that the computation over the different *k* tapes is conveniently an ‘embarrassingly parallel’ problem.

Overall, the calculation is polynomial in the size of the input, ${{{\rm{O}}}}(knN+kn{N}^{2})={{{\rm{O}}}}(kn{N}^{2})$. For a fixed $N$ and $k$, the runtime scales linearly with the number of tips in the tree, meaning the number of cells in the dataset.

### Implementation

We implement the above editing model alongside an edit sequence simulator based on the feast package^42^ and a likelihood calculation as a new package, SciPhy,– for the phylogenetic inference platform BEAST 2 (v. 2.7.8). Specifically, we introduce a new substitution model class, SciPhySubstitutionModel, which replaces standard models such as the Jukes–Cantor model to compute transition probabilities between edit states. We also extend the GenericTreeLikelihood class in BEAST 2 with the SciPhyTreeLikelihood class tailored to sequential editing. This implementation leverages domain-specific properties of lineage tracing data—for example, that the set of possible ancestral sequences is known. Upon installing the SciPhy package, these components are directly available to BEAST 2 users via both the command-line interface and the graphical interface BEAUti.

BEAST 2 performs phylogenetic inference by using Markov Chain Monte Carlo (MCMC) to quantify the posterior distribution of the lineage tree and model parameters given a set of tape sequence alignments, where each tape may be specified to have independent properties (such as tape length/truncation, and editing features). During the MCMC, tree operators modify the tree to explore all possible topologies that could explain the data. As some of these changes are local, the likelihood for subtrees that are not modified remains unchanged. We make use of that property by implementing caching, where the likelihoods for subtrees are saved at each internal node to further speed up calculations (3.5-fold speed up on a personal computer running OSX on Intel(R) Core(TM) i5 Dual-Core @2.30GHz). Additional speed-up is achieved by computing the likelihood for each tape alignment in parallel, which is easily achieved with existing components in BEAST 2 that support threading. These software optimizations lead to an overall 8-fold speed up relative to the naive implementation for an analysis involving 1000 sequences using six computing cores on the “Euler” scientific computing cluster. Using this setup, we provide realistic estimates for the average runtime required for convergence on smaller datasets from the validation study, and when varying the number of editing sites in Supplementary Figs. 1 and 2.

### Validation

We employed well-calibrated simulations^16^ to ensure the correct implementation of our Bayesian method SciPhy. We further used this simulation setup to assess the amount of information in the simulated data regarding parameters of our model and key features of lineage trees. We simulated 100 phylogenetic trees under a birth-death sampling model^28^ with parameters provided in Supplementary Table 1.

We simulated alignments of tapes along each of the 100 trees. First, we drew the rate of edit introduction (or clock rate) and edit probabilities to generate 13 distinct edits from a defined distribution (Table 2). Then, we simulated the editing process of 10 independent tapes along each phylogenetic tree under these parameters. The resulting alignments of tapes were then used as input data for SciPhy to infer the phylogenetic tree as well as the rate of edit introduction and edit probabilities, using the same distributions (i.e., Table 2) as priors. For all parameters, we used an effective sample size (ESS) value of 200 as a threshold to ensure convergence of the MCMC chains. Using these outputs, we first compared the 100 simulated (true) parameters and trees with their estimated counterparts. Specifically, we chose tree height, tree length, and tree balance as summary statistics for comparison of the lineage trees. Tree balance was calculated using the B1 index, as implemented in package TreeStat2 (v. 0.2.0)^43^. For each dataset, we additionally constructed a point estimate of the lineage tree from the sampled posterior set of trees using the conditional clade distribution (CCD) algorithm (with the CCD0 parameterization^19^) and assessed accuracy in lineage reconstruction by reporting the Phylogenetic Information (PI) distance to the true tree^18^, compared to a randomly reconstructed tree obtained by simulation under the birth-death sampling model. We test the difference between distances obtained with SciPhy and the random reconstruction using a paired *t*-test.

We chose the distributions over the editing model parameters to be representative of a broad range of editing dynamics. The clock rate distribution was selected to result in between one and eight insertions for the experiment duration. We used a Dirichlet distribution with a concentration parameter of 1.5 to draw the edit probabilities. This allows most edits to occur at similar, low probabilities, while a few edits could occur more frequently.

For each MCMC inference, SciPhy was run for at most ${10}^{9}$ iterations to estimate the clock rate, edit probabilities, and lineage tree, keeping all other parameters fixed to the truth (i.e., the value of the simulation). We used an ESS value of 200 for all parameters as a threshold to ensure convergence of the MCMC chains. We then discarded the initial 10% of the iterations as burn-in. Lastly, we calculated the coverage as the proportion of times the simulated (true) value fell within the estimated 95% HPD (highest posterior density) interval. For each of these datasets, we estimate the total runtime necessary for convergence by weighting the total empirical runtime by the ratio between the final obtained ESS and the desired minimum ESS of 200, as reported in Supplementary Fig. 1. For the first simulated lineage tree, we repeat this estimation for tapes harboring an increasing number of editing sites, as reported in Supplementary Fig. 2.

We assess the effect of alignment sparsity on the accuracy of phylogenetic reconstruction using SciPhy by generating incomplete alignments as input data for the same inference setup as used in the validation study. We consider two main mechanisms leading to tape loss: heritable loss of tapes at a constant rate through time, capturing, e.g., transgene silencing, and tape dropout at sequencing, assuming that tapes will fail to be captured by the sequencing pipeline with a given probability. We simulated the editing process with tape loss for 20 independent tapes along each phylogenetic tree, and chose wide distributions for the rate of heritable loss and dropout probability, such that we would expect between 9.7% and $88\%$ of sequenced tapes to be missing per simulated alignment (see Supplementary Table 3). This led to an average of 11 tapes recovered per cell over all simulations (see Supplementary Fig. 26). For each of these lossy tape alignments, we generate input alignments by constructing the largest complete set of tapes without missing data that includes at least a third of all cells (“Filtered lossy data”). We used these alignments to infer the phylogenetic tree for the subset of cells, as well as the editing and growth parameters, and assessed the accuracy in lineage reconstruction as described above.

### Methods benchmark

We reuse nine datasets from the validation study, sampling evenly across tree sizes by categorizing datasets into three groups: small (0–150 tips), medium (150–450 tips), and large (450–650 tips). From each category, we select three datasets to ensure balanced representation across tree sizes in our benchmark. For SciPhy, we reuse the previously inferred results from the validation study. We run the ordering-aware UPGMA as done before^10^, as well as standard UPGMA using hierarchical clustering with average linkage. TiDeTree (version 1.0.2)^14^ is run using the same prior distributions as used for SciPhy. Additionally, for each dataset, we simulate a random tree under a birth–death sampling model using TreeSim (version 2.4)^44^, conditioning on the number of tips to match that of the dataset. We report the normalized phylogenetic information distance using TreeDist (version 2.12.0)^45^.

### Phylodynamic analysis of HEK293T expansion

We use SciPhy for the analysis of the publicly available dataset (i.e., taken from https://github.com/shendurelab/DNATickerTape, Supplementary_File_2_DataTableMOI19.csv) generated as described in ref. ^10^. This study uses genetic tapes that contain 5 target sites for insertion, where each insertion step consists of the introduction of a trinucleotide character. We process the tape sequences as follows. We first retain all 3257 cells containing all 13 most frequent tapes for subsequent analysis, as done before. Out of these 13 tapes, 3 (barcoded with ‘TTCCGTCA’, ‘TGGTTTTG’, and ‘TTCACGTA’) are found to possess fewer than the five original editable target sites, respectively four (‘TTCCGTCA’, ‘TGGTTTTG’) and two (‘TTCACGTA’) sites. We refer to these tapes as “truncated”. This procedure yields a dataset containing 19 different possible trinucleotide insertions. We create three random subsets of this data, each consisting of 1000 cells, and perform phylodynamic analysis on each subset.

We use SciPhy to model the editing dynamics in this filtered dataset. We assume that each of the 13 tapes can accumulate edits at an independent rate, while insertion probabilities are shared across all tapes. We place prior distributions on the parameters of the editing model as described in Table 3. As no quantitative information on the editing dynamics is available for this experimental setup, we use broad priors that capture a variety of experimentally plausible values (as in the validation part). For example, the clock rate prior spans between one and ten edits per tape over 25 days. As each tape has a maximum of five sites, ten edits correspond to the scenario of early saturation. The prior is centered on 5, the desired edit count. The prior on the edit probabilities is a Dirichlet prior with concentration 1.5, chosen to reflect expected asymmetries in editing outcomes observed in similar non-sequential recorders^46^.

**Table 3:** Priors used for analysis of the HEK293T dataset using SciPhy and the birth-death sampling model

Parameter	Prior distribution	95% HDI
Edit probabilities	Dirichlet (1.5)	[1.3e-4, 1.3e-1]
Clock rate	Log-normal (−2, 0.5)	[3.5e-2, 3.1e-1]
Birth rate	Log-normal (−0.6, 1)	[0.1, 2.8]
Death rate	Log-normal (−2, 1)	[3.5e-3, 0.7]
Sampling proportion	0.0008	fixed
Sampling proportion	Log-normal (−7.4, 1.2)	[4.8e-6, 4.4e-3]
Origin or experiment duration	25	fixed

Being sampled from a monoclonal culture, cells are expected to undergo exponential growth and to not acquire any specific population structure. Consequently, we choose to infer the dynamics underlying the growth of this cell population using the birth–death sampling model for constant and piecewise constant rates^28^. We set priors on the birth and death rate (Table 3) that are very broad, leading, for instance, to between 12 and $2.5 \times {10}^{30}$ cells at the time of sequencing, which broadly contains the reported number of cells ($1.2 \times {10}^{6}$). We additionally input known experimental parameters by setting the origin time to the experiment duration of 25 days and fixing the sampling proportion to $1000/(1.2 \times 10^6)=0.0008$. In the piecewise constant analysis, rates were allowed to vary every 2 days, yielding a timeline of 13 epochs where an Ornstein–Uhlenbeck (OU) prior is applied to the birth and death rates, and where we apply a Log-normal (0.5, 0) hyperprior on parameters $\sigma$ and $\nu$ of the OU process.

For each subset of the data, we run three MCMC chains for at most ${10}^{9}$ iterations. We assess convergence by confirming that the three chains are sampling the same stationary distribution, with an ESS value per chain $>$200 for all parameters of interest and visually in Tracer (v. 1.7.2)^15^. We discard 10% burn-in and combine all three chains using LogCombiner (v. 2.7.7)^15^. The combined ESS and $\hat{r}$ values for the posterior probability of all analyses of this dataset are reported in Supplementary Table 4.

To test whether the ordering of 4× and 5× tapes is unexpected under the assumption of equal editing rates for all tapes, we compute the probability of the observed (i.e., that *k* of the three tapes with the highest editing rates are 4× tapes, one-sided test) using the hypergeometric distribution. We model the setup as follows: We have a total of 12 tapes (*N* = 12), of which three tapes (*K* = 3) have four sites and nine tapes have five sites. If we draw the three tapes with the highest rates (*n* = 3), we want to calculate $p(k)$, the probability that *k* of these tapes are of type 4×. We do this using the standard hypergeometric distribution:

$$p(k)=\frac{\left({K}\atop{k}\right)\left({N-K}\atop{n-k}\right)}{\left({N}\atop{n}\right)}$$

To compare the UPGMA tree to SciPhy’s estimated trees on real data, we use the R package TreeDist (v. 2.12.0)^45^ to provide mappings of tree sets for four dimensions, pairwise. For this, we extract a random subsample of 1641 trees inferred under SciPhy to calculate distances to the UPGMA topology. We do so using three metrics for topological comparisons, namely the Robinson–Foulds distance, the Phylogenetic Information distance, and the Clustering Information distance. Further, we employ one metric that also incorporates differences in branch lengths, namely the weighted Robinson–Foulds metric. For each metric, we perform multidimensional scaling to map these distances in lower dimensions using principal coordinate analysis^47^. Out of 12-dimensional mappings obtained we selected the number of dimensions required to faithfully visualize tree distances based on the product of the trustworthiness and continuity metrics as described by Smith et al.^45^. For all mappings, we find that four or more dimensions provide acceptable levels of distortion (trustworthiness × continuity > 8.5). These first four dimensions, which we label 1–4 in our plots, are used for reference but do not represent interpretable features of analyzed trees.

### Gastruloid experiment

#### Culturing and genetic engineering of the mouse embryonic stem cells (mESCs)

We adopted a standard protocol for culturing mESCs^48^, which is described in detail in Regalado, Qiu et al.^49^. The mESCs (E14TG2a) were kindly provided by Schröter. Briefly, mESCs were cultured on a gelatin-coated plate using the Serum-based medium, which is GMEM (Gibco) supplemented with 8% fetal bovine serum (FBS, Biowest), 8% KnockOut Serum Replacement (KSR, Gibco), 1× Glutamax (Gibco), 1× MEM non-essential amino acids (Gibco), 1 mM Sodium Pyruvate (Gibco), and 0.1 mM beta-mercaptoethanol (BME, Gibco). Gelatin-coated plates were prepared by making 0.2% (w/v) gelatin solution (Sigma, G1393) and autoclaving the bottle, which was applied to each culturing well (1 mL per well within a 6-well culturing plate) and incubated in a tissue culture incubator set to 37 °C with 5% CO_2_ for at least 30 min. After the coating, the gelatin solution was aspirated from the plate immediately before depositing mESCs. Cells are grown in the stem-cell-designated incubator (set to 37 °C with 5% CO_2_) and biosafety cabinet to avoid cross-contamination.

To allow DNA Typewriter-based lineage recording, mESCs were genetically engineered with the piggyBac transposition system that integrated: (1) PB-TAPE, a transposon library including TAPE-targeting epegRNAs (random NNNGGA insertions) expressed from the U6 promoter and GFP transcript including a DNA Tape sequence in its 3′-UTR, (2) PB-PEmax, a transposon including Prime Editor (PEmax) expression cassette with constitutively expressed Puromycin resistance gene (PuroR), and (3) PB-mCherry, a transposon that expresses mCherry as a high-integration selection carrier. DNA Tape is structured to include the T7 promoter sequence, a 12-bp Tape-specific degenerate barcode (TapeBC, NNNNNAANNNNN), 6xTAPE capable of six sequential insertions, and 10× Genomics Capture Sequence 1 (CS1) to aid recovery of DNA Tape during scRNA-seq.

These transposon vectors and transposase plasmids were mixed with the 73:20:2:5 mass ratio (PB-TAPE:PB-iPEmax:PB-mCherry:Super piggyBAC transposase expression vector, SBI), then transfected using Lipofectamine 2000 (ThermoFisher) protocol (4 µg DNA per 1 million cells in 6-well plate). Transfected cells were cultured for 5 days and selected under 2 µg/mL Puromycin for another 5 days to ensure integration of PB-iPEmax. Selected cells were dissociated into single cells and flow-sorted by mCherry expression (from PB-mCherry), then plated at low seeding density for monoclonal gastruloid induction.

#### Monoclonal gastruloid induction and single-cell RNA-seq profiling

We used the monoclonal gastruloid induction protocol, as described in ref. ^49^. Briefly, we cultured genetically engineered mESCs on a layer of Mouse Embryonic Fibroblast (MEF) cells at a low seeding density, resulting in the formation of monoclonal colonies across the culturing plate after 5 days. On Day 4, the mESC colonies were selectively removed from the MEF layer using Collagenase IV treatment and transferred to a non-adherent 10-cm plate in the differentiation medium (NDiff227, Takara), spontaneously forming 3D spheroids. After 24 h, 3D spheroids or aggregates were manually picked into 96-well plates (non-adherent, round bottom) and subsequently used for the downstream gastruloid induction protocol^50^, including pulsing with 3 µM CHIR-99021, subsequently described as CHIR pulse, for 24 h, followed by 3 days of culturing in NDiff227 medium until the harvesting of gastruloids for single-cell profiling (dissociated and processed following the conventional protocols: Cell Preparation guide, CG00053 Rev C, and User Guide for Chromium Next GEM Single Cell 3′ HT Reagent Kits v3.1, Rev D). Resulting libraries are sequenced on NextSeq2000 (Illumina).

Next, we processed the sequencing reads of the DNA Tape captured in the scRNA-seq experiment. The single-cell transcriptome data were processed using Cell Ranger 7.2.0^51^ with default settings (e.g., --include-introns true) and refdata-cellranger-mm10-3.0.0 as the reference. We extracted cell-specific barcode (CellBC), UMI added during cDNA synthesis, TapeBC, and 6 InsertBC from each DNA Tape sequencing read, and removed reads with less than 2 UMI associated with particular CellBC-TapeBC-6xInsertBC combinations. In the resulting reads, if there were multiple 6xInsertBC read patterns recovered per CellBC-TapeBC combinations, 6xInsertBC with the highest read counts were retained, resulting in a table of CellBC, TapeBC, and 6xInsertBC sequences. This table was used for the lineage tree reconstruction.

### Gastruloid analysis

We use SciPhy to analyze DNA Typewriter tapes derived from the experiment described above. Similar to the analysis of the HEK293T dataset, the full set of sequenced cells was filtered for cells where eight of the most frequent tapes could be recovered by sequencing. This process yielded a set of 780 cells retained for our phylodynamic analysis, where 42 different trinucleotide characters were found to have been inserted into the tapes. We use SciPhy to model the prime editing process throughout the experiment for the DNA Typewriter tapes, where, as previously, each of the eight tapes acquires edits at an independent rate, but where insertion probabilities for the 42 trinucleotide characters are shared across the tapes. We use the same prior distribution on the insertion probabilities as in the cell culture analysis, based on the expectation that editing outcomes are not uniformly distributed. For the clock rate, we adjust the prior to reflect the shorter 11-day time frame of the gastruloid experiment. The 95% highest density interval again corresponds to an expected range of 1–10 edits per tape, with the median aligned with the desired outcome of five edits (see Table 4 for prior distributions).

**Table 4:** Priors used for the analysis of the gastruloid dataset using SciPhy and the birth-death sampling model

Parameter	Prior distribution	95% HDI
Edit probabilities	Dirichlet (1.5)	[1.2e-4, 1.3e-1]
Clock rate	Log-normal (−1.1, 0.8)	[2.4e-2, 1.2]
Birth rate	Log-normal (0.1, 1)	[2.8e-1, 5.7]
Death rate	Log-normal (−0.4, 1)	[1.7e-1, 3.5]
Sampling proportion	0.0195	fixed
Origin or experiment duration	11	fixed

We study the growth dynamics that drive the formation of this gastruloid by fitting a birth-death sampling model with piecewise constant rates. We hypothesized that the dynamics that underlie gastruloid formation are predominantly driven by experimental steps that lead to (1) aggregate formation, (2) aggregate growth, (3) Chiron-pulse driven (4) gastruloid elongation. Therefore, we allow birth and death rates to vary between these experimental steps, leading to two variants of the analysis:

1. Variant 1: considers birth and death rate changes between the steps of aggregate formation and gastruloid elongation only, leading to two change points, at days 4 and 7.5, shown in the main text, Fig. 5.
2. Variant 2: considers birth and death rate changes between all steps of gastruloid growth, including the CHIR pulse, leading to three change points at days 4, 7, and 8, shown in Supplementary Fig. 18.

We set broad priors on the birth and death rate, consistent with a growth leading to up to $\sim {10}^{7}$ cells, and additionally fix the sampling proportion and origin time as known experimental parameters. Rate variation between different epochs of the experimental timeline in both variants of the analysis was modeled using an Ornstein–Uhlenbeck (OU) with a Log-normal (0.5, 0) hyperprior on parameters $\sigma$ and $\nu$ of the OU process. All detailed priors are listed in Table 4.

The inference was performed as follows: we ran three MCMC chains on the dataset for at least $\sim {10}^{9}$ iterations or until convergence. We assessed convergence and combined all three chains as previously described (see above). The combined ESS and $\hat{r}$ values for the posterior probability for our analysis of this dataset are reported in Supplementary Table 4.

To evaluate our estimates of the growth through time against empirical measurements, we use a time series of the total cell count for gastruloids reported in ref. ^21^ (Extended Data Fig. 2b). We calibrated their experimental timeline to match ours by aligning the day of CHIR application with day 7. Assuming exponential growth through time (as previously done in ref. ^21^), we calculate the growth rate in each time interval ${t}_{d}$ (in days) as follows. Given a cell count ${N}_{0}$ at time ${t}_{0}$ and ${N}_{1}$ at time ${t}_{1}={t}_{0}+{t}_{d}$, the growth rate ${g}_{d}$ on the interval ${t}_{d}$ is expressed as:

$${g}_{d}=\frac{1}{{t}_{d}}\log \left(\frac{{N}_{1}}{{N}_{0}}\right)$$

For comparison to our estimates, we calculate the growth rate over time periods that most closely match our chosen intervals for inference. For example, in Fig. 5D, the rates are calculated between day 7 and day 10 to match our last time period, between 7.5 and 11 days.

### Reporting summary

Further information on research design is available in the Nature Portfolio Reporting Summary linked to this article.

## Supplementary information

Supplementary Information

Reporting Summary

Transparent Peer Review file

## Source data

Source data

## Data Availability

Raw sequencing data and associated processed data for the monoclonal gastruloid analysis have been uploaded to the Gene Expression Omnibus (GEO) and are available under accession number GSE315827 [https://www.ncbi.nlm.nih.gov/geo/query/acc.cgi?acc=GSE315827]. The processed data underlying the analysis of the cell culture (HEK293T) data is publicly available and was taken from https://github.com/shendurelab/DNATickerTape, Supplementary_File_2_DataTableMOI19.csv. Source data are deposited at https://github.com/seidels/sciphy-materials/releases/tag/release-source-data. Source data are provided with this paper.

## Code availability

All code to reproduce the analyses is publicly available from https://github.com/seidels/sciphy-materials. The SciPhy codebase is openly accessible at https://github.com/azwaans/SciPhy.
