# Supplementary material for: SciPhy: A Bayesian phylogenetic framework using sequential genetic lineage tracing data
Source: Nat Commun. 2026 Jun 10;17:7398. doi: 10.1038/s41467-026-73377-6 (PMC13402801; doi:10.1038/s41467-026-73377-6)
Supplement: Supplementary file 3 — Transparent Peer Review file [file 41467_2026_73377_MOESM3_ESM.pdf]

nature portfolio

Peer Review File

SciPhy: A Bayesian phylogenetic framework using sequential genetic lineage tracing data

Corresponding Author: Dr Sophie Seidel

This file contains all reviewer reports in order by version, followed by all author rebuttals in order by version.

Version 0:

Reviewer comments:

Reviewer #1

(Remarks to the Author)
Seidel and Zwaans et al introduced a Bayesian phylogenetic approach, SciPhy, to estimate time-scaled cell phylogenies and population dynamics from sequential insertion-based editing lineage tracing data. They applied SciPhy to simulation data and lineage tracing data from a monoclonal culture of HEK293T cells and gastruloid derived from a single mESC. Results showed that SciPhy provides more accurate inference of cell lineage tree while reporting uncertainty and proliferation rates. I have some suggestions for improvement.

Major Comments:
1. I acknowledge that using sequential insertion mutations indeed helps refine phylogenetic inference. However, obtaining such lineage tracing data with the DNA Typewriter system is technically challenging. The authors should discuss whether the method is also suitable for other lineage tracing methods with dynamic DNA barcoding.

2. The authors demonstrate the advantages of the SciPhy method by comparing it with UPGMA. However, I believe the authors should also compare it with other methods in BEAST which do not utilize the property of sequential mutations to illustrate the importance of sequential mutations for phylogenetic inference.

3. In in-silico simulations, the authors demonstrate the accuracy of SciPhy's inference by comparing editing rates and insertion probabilities. However, these are not all the parameters inferred by SciPhy, and there is also a lack of comparison between the ground-truth lineage tree and the SciPhy reconstructed tree. Additionally, there is a lack of model robustness analysis (mutation rate, cell number, sampling proportion, mutation sparsity, etc.).

4. I have some concerns regarding SciPhy's computational performance. The authors claim that the analysis of 1000 cells require ~15 days, and the computation time grows exponentially, which is generally unacceptable. I was wondering what sampler the authors used for MCMC sampling. Perhaps considering the currently commonly used No-U-Turn Sampler (NUTS) (arXiv:1111.4246) could accelerate sampling.

5. Additionally, the authors mentioned the convergence issue of the SciPhy method. The authors should show the convergence statistics for each parameter in each dataset in the study, such as rhat and/or other statistics. For this issue, non-convergence of large datasets might be due to the high correlation between parameters, and the Differential-Evolution MCMC method (arXiv:1710.09486) might solve this problem.

Minor Comments:
In Figure 4a, it is unclear what the four coordinates for the RF distance comparison represent.

The probability transition model in section 4.2 is the core part of the SciPhy model. However, this section is not very clearly written, with confusing symbol usage in eqn(1)-eqn(3) and a lack of detailed explanations.

(Remarks on code availability)

Reviewer #2

(Remarks to the Author)
The authors developed a mechanistic model of sequential genome editing, SciPhy, within the BEAST 2 framework, allowing for estimation of time-scaled phylogenies and cell population parameters.
SciPhy was validated using simulations and applied to two real datasets:
a) A monoclonal culture of HEK293T cells, where SciPhy estimated editing dynamics, cell lineage trees, and population growth rates over a 25-day period.
b) Murine gastruloids modeling early mammalian development, in which SciPhy analyzed complex growth dynamics during gastruloid formation, including changes in growth rates following CHIR treatment.
Overall, the model formulation and inference appeared to be solid. However, there remains unanswered questions from the manuscript in terms of its application to real datasets. We raise the following major issues:
1. The most important result for time-scaled cell phylogeny inference is the cell tree. Because applications to the experimental data have no ground truth, the validation of the methodology and its accuracy under different circumstances is highly dependent on the simulation study. However, the simulation study presented here is inadequate. The author did not provide any results regarding the cell tree accuracies in the simulation study, only the model parameters were compared. It is necessary to compare the performance in both topology distance and branch length error. We suggest the following:
a. The proposed method should be compared to UPGMA in terms of tree reconstruction accuracy under different parameters.
b. The performance trend should be explored with respect to different editing parameters.
The results are necessary for evaluating the number of errors and advancement there is in terms of reconstruction accuracy.
2. One major concern in Bayesian inference applied to cell lineage tracing data is scalability, especially the performance with many cells. We think a scalability analysis is necessary. The author should apply the methods to simulated datasets of varying number of cells and sites, and quantify trend of runtime, as well as the performance in terms tree reconstruction error at different running time. Regarding this point, the author has made qualitative statements such as “MCMC methods searching tree space may not converge for trees with more than around 1000 tips”. A quantitative analysis of the scalability of the method is necessary, including the conditions under which the method will not converge, etc.
3. Adding to the last two points, the simulation study can and should also be used to evaluate the exact experimental scenarios in which the method was applied later in the manuscript to offer an idea of the amount of error in this estimation. For example, what is the average error in the inferred time of divergence in both experiments?
4. In the HEK293T analysis, the author suggested that the early and late edits may have different rates, but it is unclear if it can be concluded that there is no difference by limiting the data to just the first two edits since the model is mis-specified. The robustness of the estimates under the scenario where early and late edits have different rates can again be tested by simulating data where the early edits are faster and applying the wrong model. One solution is to relax the uniform editing rate assumption for early and late estimates.
We think this work can also be much improved by addressing in addition the suggestions below:
1. One natural question regarding the lineage tracing using a sequential recorder is how this system compares to a more traditional recorder without sequential information. As the authors have also previously done work on traditional recorders. It will be natural and highly valuable to provide a comparison of the two under comparable number of recording sites and rates.
2. The implementation of the model is heavily reliant on the BEAST2 framework, but the details on how this model is implemented within BEAST2 are not very clearly described. As people in cell biology may not be familiar with the framework, some details should be presented that are approachable by someone unfamiliar with BEAST2. For example, which components of BEAST2 have been modified in which way.
3. The branch length estimates are expected to be better if more accurate prior information on editing rates is available. Similarly, tree inference should improve given prior estimates on insert probabilities. Generally, these parameters are not hard to measure experimentally for a given system. The author can demonstrate the effect of having prior on these and the improvements that can be made in simulation.

(Remarks on code availability)

Reviewer #3

(Remarks to the Author)
Seidel et al. introduce SciPhy, a Bayesian phylogenetic framework for reconstructing time-scaled lineage trees and inferring cell population dynamics using CRISPR-based sequential genetic lineage tracing data. They integrate the framework into the BEAST 2 platform, enabling the analysis of sequential genome editing data that tracks not only genetic edits but also the order of their accumulation. SciPhy was validated through simulations and applied to real-world datasets, including lineage tracing data from HEK293T cells and murine gastruloids. Compared to traditional clustering methods like UPGMA, SciPhy demonstrates improved accuracy in tree topology and allows for the simultaneous inference of parameters such as cell division rates, editing rates, and sampling proportions. Specific strengths include its capacity to handle uncertainty in lineage relationships and its ability to estimate time-resolved growth dynamics, which is particularly advantageous for datasets with dynamic lineage tracing.

The manuscript was well written and here are some suggestions that may enhance the manuscript:

* The manuscript does not provide sufficient discussion on how SciPhy handles missing or noisy lineage tracing data, which

is common in CRISPR-based lineage tracing due to dropout, sequencing errors, or incomplete editing. Missing data can skew tree reconstruction, particularly when certain edits or cells are underrepresented, leading to biased estimates of population parameters. The authors need to implement a model that explicitly accounts for dropout and sequencing errors, as seen in other tools like the PMM model in Mai et al. 2024. The authors need to add simulation studies to assess SciPhy's robustness under varying levels of data noise and missingness. The authors should also include a discussion on limitations due to missing data and the effect on parameter inferences.

* Bayesian inference in SciPhy involves computationally intensive MCMC methods, which may become infeasible for datasets with thousands of cells. This will severely limit SciPhy's application to larger lineage tracing datasets, particularly those involving whole-organism or long-term lineage studies. I would like to see the authors to explore parallelized or approximate inference methods, such as variational inference or Bayesian posterior approximations, including benchmarks comparing SciPhy's performance on large datasets with other tools like UPGMA and GAPML.

* The dependence on priors for editing rates, insertion probabilities, and population parameters was not thoroughly validated against empirical data. Over-reliance on priors may bias results, particularly if the priors are poorly chosen or do not reflect experimental realities. I would like to see sensitivity analyses to evaluate the impact of different prior distributions and use empirical datasets to justify the choice of priors and refine them accordingly.

(Remarks on code availability)

Version 1:

Reviewer comments:

Reviewer #1

(Remarks to the Author)
My concerns have been well addressed by the authors. I have no additional comments.

(Remarks on code availability)

Reviewer #2

(Remarks to the Author)
The authors have addressed many concerns and comments from the initial review, including more simulation validation, benchmarking against alternative inference methods, and improved clarity of model and BEAST2 implementation. These updates strengthen the paper. However, two important points remain that would improve the completeness of the manuscript:
1. While the authors provided a detailed runtime analysis as a function of the number of cells, scalability with respect to the number of editing sites remains untested. Understanding scaling performance with number of sites is valuable theoretically for the readers and important for guiding future applications. The author should test the scaling at intermediate cell counts and increasing site numbers.

2. We appreciate that a methodological extension for dropout modeling could be the subject of a separate manuscript. However, simulations incorporating dropout and sequencing noise fall within the scope of this study. The current model should be stress-tested under realistic experimental error scenarios (e.g., missing tapes, truncated barcodes, sequencing errors), particularly because these issues are relevant in the datasets analyzed. Including a simulation with different levels of noise is essential for this work.

(Remarks on code availability)
A basic tutorial on the code is present.

Reviewer #3

(Remarks to the Author)

1. Robustness to dropout/error
The authors acknowledge that explicit modeling of dropout/sequencing error is a current gap and describe an in-progress likelihood extension, with added discussion on bias from lossy data. However, no quantitative stress-tests are provided. While this may be expanded in a separate study, inclusion of a focused robustness analysis in the current manuscript is essential for practical applicability. Please add a compact simulation (Supplement is fine) varying tape-level dropout and readout/assignment error, reporting effects on topology (e.g., PI, wRF), branch-length accuracy, and growth-rate bias/coverage—ideally with a minimal implementation of the referenced extension and a brief with/without ablation.

2. Scalability — acceptable and transparent.
The authors clearly state practical limits (~1000 cells; ~15 days for the empirical run), explain that likelihood scales linearly while tree-space exploration is the bottleneck, and report convergence metrics (ESS targets, r-hat) and runtime trends. This is sufficiently transparent; no further changes requested.

(Remarks on code availability)

Version 2:

Reviewer comments:

Reviewer #2

(Remarks to the Author)
The authors have addressed the remaining concerns raised in the previous round of review. I have no further comments.

(Remarks on code availability)

Reviewer #3

(Remarks to the Author)
The authors have addressed all my requests comprehensively. The manuscript is good for publication.

(Remarks on code availability)

Open Access This Peer Review File is licensed under a Creative Commons Attribution 4.0 International License, which permits use, sharing, adaptation, distribution and reproduction in any medium or format, as long as you give appropriate credit to the original author(s) and the source, provide a link to the Creative Commons license, and indicate if changes were made.
In cases where reviewers are anonymous, credit should be given to 'Anonymous Referee' and the source.
The images or other third party material in this Peer Review File are included in the article’s Creative Commons license, unless indicated otherwise in a credit line to the material. If material is not included in the article’s Creative Commons license and your intended use is not permitted by statutory regulation or exceeds the permitted use, you will need to obtain permission directly from the copyright holder.
To view a copy of this license, visit https://creativecommons.org/licenses/by/4.0/

POINT BY POINT RESPONSE

We provide the replies in dark blue. Text quotes from the edited manuscript are *italicised*, new text is underlined, deleted text is ~~struck-through.~~

REVIEWER COMMENTS

Reviewer #1 (Remarks to the Author):

Seidel and Zwaans et al introduced a Bayesian phylogenetic approach, SciPhy, to estimate time-scaled cell phylogenies and population dynamics from sequential insertion-based editing lineage tracing data. They applied SciPhy to simulation data and lineage tracing data from a monoclonal culture of HEK293T cells and gastruloid derived from a single mESC. Results showed that SciPhy provides more accurate inference of cell lineage tree while reporting uncertainty and proliferation rates.

We thank the reviewer for the constructive summary of our work and their recognition of the strengths of SciPhy in accurately inferring cell lineage trees, reporting uncertainty, and estimating proliferation dynamics.

I have some suggestions for improvement.

Major Comments:

1. I acknowledge that using sequential insertion mutations indeed helps refine phylogenetic inference. However, obtaining such lineage tracing data with the DNA Typewriter system is technically challenging. The authors should discuss whether the method is also suitable for other lineage tracing methods with dynamic DNA barcoding.

We thank the reviewer for prompting us to consider the broader applicability of SciPhy. In principle, SciPhy is designed for lineage tracing data generated by *DNA Typewriter* and *peChyron*, as well as potential future methods derived from these approaches. The key requirement for SciPhy's applicability is that the barcoding system generates sequential edits, where the order of edit accumulation is known.

For lineage tracing systems with random edits, alternative methods may be more appropriate. For example, *TiDeTree* (Seidel & Stadler 2022) can be used if the edits occur at scattered sites and each edit affects only a single target site, while *GABI* (Zwaans et al. 2025) is suitable when edits occur in a contiguous array and a long deletion at one site can influence adjacent sites.

While this specificity may seem like a limitation, we argue that it is, in fact, a strength. By explicitly modeling the known editing process, SciPhy incorporates additional biological information that enhances inferential power. In contrast, a model designed to accommodate any substitution process would lack this information and thus have reduced inferential power.

2. The authors demonstrate the advantages of the SciPhy method by comparing it with UPGMA. However, I believe the authors should also compare it with other methods in BEAST which do not utilize the property of sequential mutations to illustrate the importance of sequential mutations for phylogenetic inference.

We agree that this is an important consideration: we have extended our benchmark to include additional phylogenetic inference methods (TiDeTree from BEAST and UPGMA without ordering) that do not leverage the known sequential order of mutations. Specifically, we reused 9 simulated datasets from our validation benchmark and assessed tree reconstruction accuracy in terms of topological error (see Methods section "Methods benchmark" for details). We have added the following text to the Results section. We also replicate updated Figure 4 D and E below.

We compare SciPhy with existing methods for phylogenetic reconstruction on simulated and real data (Fig. 4). For the comparison on simulated data, we reuse a subset of 9 datasets from the validation study (see Methods for details). We contrast SciPhy with current approaches that used order-aware UPGMA clustering. We also compared against methods that do not use the ordering information from the DNA Typewriter data, namely TiDeTree and UPGMA clustering based on an order-unaware distance matrix. As a baseline, we included random trees sampled from a birth–death model with the same number of tips.

We find that SciPhy's tree point estimate based on the conditional clade distribution (CCD) achieved the lowest median Phylogenetic Information (PI) distance, indicating the highest reconstruction accuracy across methods. Notably, TiDeTree performed comparatively well despite not having access to the true order of edit acquisition, highlighting its ability to infer ordering from the data in many cases. However, we expect the performance gap between any method only modelling unordered edits and SciPhy to widen in scenarios where the edit distribution is more skewed—i.e. where some edits are much more frequent than others—since having access to the true order of edits becomes increasingly informative in such settings.

To evaluate the combined performance of tree topology and branch length estimation, we compared the estimated trees in terms of the weighted Robinson Foulds distance (wRF, (Fig 4). Here, SciPhy clearly outperforms all other methods. This advantage arises from SciPhy's explicit model of sequential edit acquisition within a tape. By contrast, both TiDeTree and unordered UPGMA treat all sites as independent and are thus misspecified. Ordered UPGMA incorporates site ordering in the distance matrix calculation (i.e. a higher similarity comparing sites within tape sites is only achieved if the previous sites are identical). However, UPGMA clustering only uses pairwise-distances and does not use the known ancestral state of each tape, nor uses the knowledge of the editing process to inform branch length inference as SciPhy does.

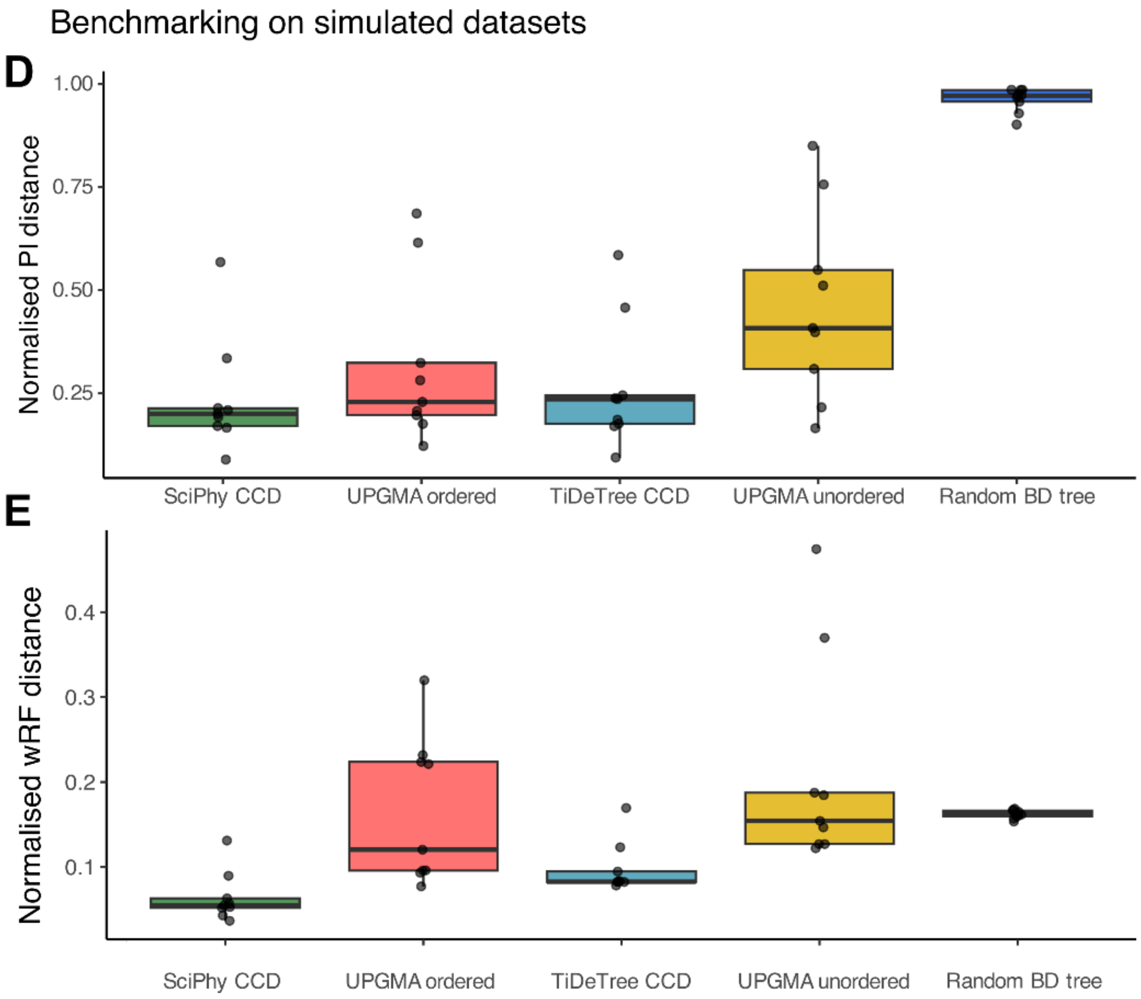

Figure 4. In D and E we extend this comparison using simulated data, and additionally compare SciPhy to counterpart methods that do not model ordered editing, i.e. TideTree,and standard, order-unaware UPGMA ('UPGMA unordered').

3. In in-silico simulations, the authors demonstrate the accuracy of SciPhy's inference by comparing editing rates and insertion probabilities. However, these are not all the parameters inferred by SciPhy, and there is also a lack of comparison between the ground-truth lineage tree and the SciPhy reconstructed tree. Additionally, there is a lack of model robustness analysis (mutation rate, cell number, sampling proportion, mutation sparsity, etc.).

We agree that it is of great interest to examine the accuracy of SciPhy with respect to lineage tree inference. We so far assumed that that components that enable this inference in BEAST2 (such as operators, tree objects, and tree priors) require no further validation. To rule out the possibility of any of these components not working sufficiently after adjusting the tree likelihood, we extended our in-silico validation study to showcase SciPhy's ability to recover lineage trees from the data. To this end, we used the same 100 datasets simulated

for validation as input for joint tree and editing parameter inference, instead of editing parameters alone originally. Specifically, we showcase SciPhy's performance for 3 summary statistics capturing complementary features of lineage trees the tree height (capturing the inferred timing for the MRCA of the sampled set of cell), the total tree length (representing the total amount of evolution in the lineage tree) as well as the tree balance (as the B1 index, capturing the shape of the tree). These 3 summary statistics could all be correctly inferred using SciPhy, as demonstrated in updated Figure 2 C-E.

As suggested, we also now compare SciPhy reconstructed trees to the ground-truth trees by measuring their phylogenetic distance and show that for the parameter regimes under which we simulated data, SciPhy is significantly able to learn lineage relationships from the data, and generally correctly reconstructs more than 50% of the phylogenetic relationships from barcode alignments (now shown in updated Figure 2 F). We further expand this exploration in Appendix Figure 2, where we show trends in reconstruction accuracy for bins of parameter values, and see that performance is most affected by the editing rate, where higher editing rates, expectedly, generate higher information content and result in better accuracy. The relative gain in accuracy w.r.t. other reconstruction methods are also addressed in our new benchmarking results (see Fig 4), where we show that SciPhy generally reconstructs more accurate trees than UPGMA, and most markedly when comparing accuracy for branch length estimation.

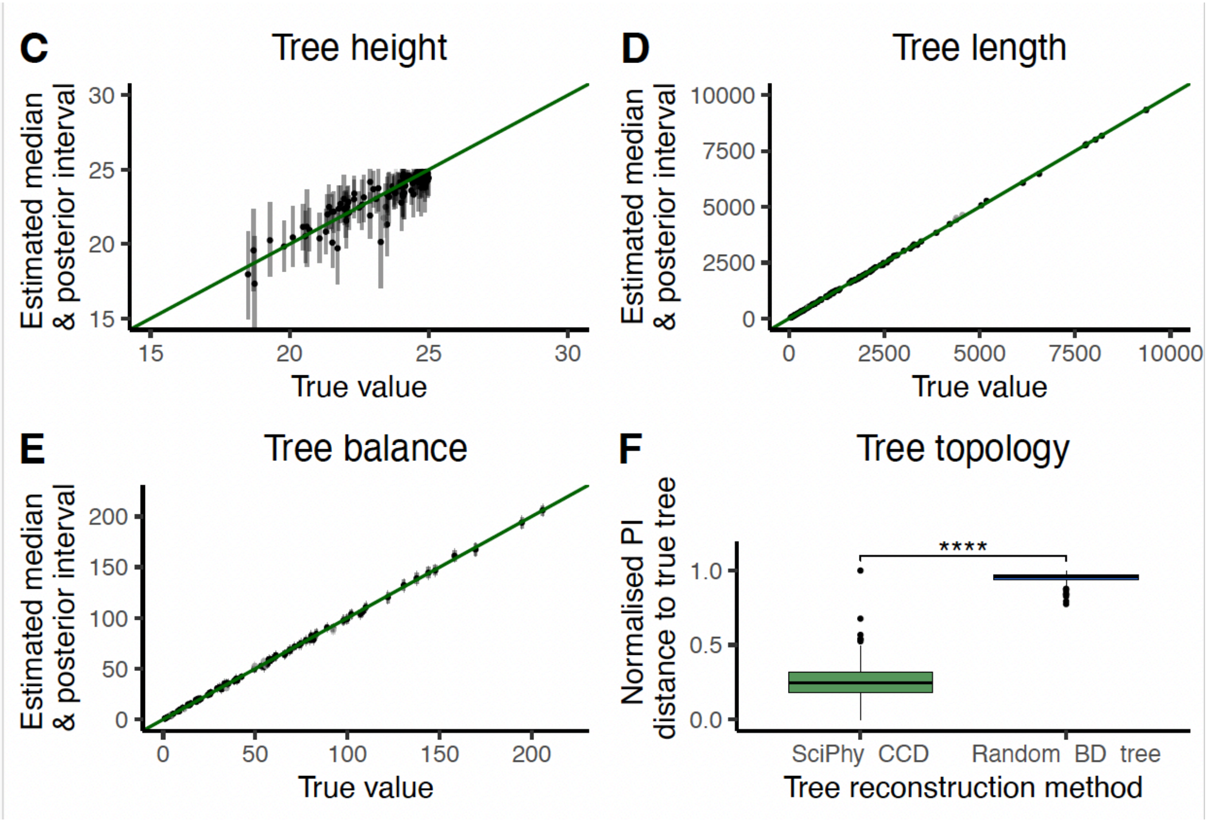

Figure 2: *We report the results of the in-silico validation for the editing rate (A), the insertion probabilities (B), tree height (C), tree length (D), tree balance ((E), calculated using the B1 index) and tree topology (F) based on 100 simulations. In all panels A-E, we plot the estimated posterior medians against the true values. The grey intervals denote the 95\% highest posterior density interval around the median. Estimated median posterior are plotted*

as black dots when the 95\% highest posterior density interval contains the true values and as grey dots otherwise. The green line represents the performance of an ideal estimator. Panel F shows the topological distance (measured using the Phylogenetic Information metric) to the ground truth simulated tree with trees inferred with SciPhy or obtained by simulations under a simple birth-death model (Random BD tree) , and annotated with the significance of a paired t-test between both methods.

The results section was adapted accordingly, as follows:

We validated SciPhy using well-calibrated simulations Dawid (1982) to ensure that it draws samples from the correct posterior distribution. This process involved simulating trees and edited barcodes for each cell, and using them as input data to estimate back the lineage tree as well as the editing parameters under the SciPhy model. Editing parameters were drawn from distributions representing realistic editing dynamics. The same distributions were also used as priors in the inference (see Methods for details). Correct tree inference was assessed through three key summary statistics of inferred lineage trees, namely, the tree height, tree length and tree balance, covering the properties of branch lengths and tree shape.

[...]

To evaluate the inference power across a range of parameter values, we plotted the simulated (true) parameters against the estimated parameters (Fig. 2 A-E). The correlations for the editing rate and the insertion probabilities (with a Pearson's R value of 0.99 and 0.98, respectively, see Appendix table 1 for 95% confidence intervals) confirm that SciPhy extracts meaningful signal from the data. *Likewise, a high correlation for all three tree summary statistics is achieved (for tree height, tree length and tree balance, Pearson's R of 0.90,0.99 and 0.99 respectively), showing that signal for these features is also derived from the data.*

We also assess SciPhy's ability to extract signal for lineage relationships from the data by comparing topological distances (using the Phylogenetic Information (PI) distance) between randomly constructed trees as well as SciPhy's tree estimates (summarised as point estimated with the conditional clade distribution (CCD)), and the true simulated topologies. SciPhy consistently and significantly recovers topologies closer to the ground truth, thus confirming its ability to learn lineage relationships from this input data (Fig. 2 F).

4. I have some concerns regarding SciPhy's computational performance. The authors claim that the analysis of 1000 cells require ~15 days, and the computation time grows exponentially, which is generally unacceptable. I was wondering what sampler the authors used for MCMC sampling. Perhaps considering the currently commonly used No-U-Turn Sampler (NUTS) (arXiv:1111.4246) could accelerate sampling.

We thank the reviewer for this comment and for suggesting the No-U-Turn Sampler (NUTS). We have investigated the use of NUTS in the context of phylogenetic inference and, to the best of our knowledge, its application has so far been limited to sampling continuous parameters, such as correlations between mixed-type traits [Suchard et al., 2023], but not to

inferring phylogenies themselves. This is likely due to the composite nature of tree space, which has both a discrete component (tree topology) and a continuous component (branch lengths). Developing differentiable representations of tree space to enable gradient-based inference remains an open research problem [Matsen et al., 2024]. Therefore, while NUTS offers advantages in continuous settings, its application to improve runtime in SciPhy is currently not feasible and beyond the scope of this manuscript.

As we note in the Discussion, the current implementation of SciPhy supports datasets up to the order of 1000 cells, with the empirical dataset taking ~15 days to converge. This is due to the exponential growth of tree space, even though **the individual likelihood evaluations scale linearly with the number of tips.** We have implemented caching and multi-threading to accelerate likelihood computation, but runtime remains a challenge. Looking forward, we see promising directions to address this. In particular, recent developments based on mutation annotated trees [Sabeti et al. 2025] have demonstrated scalability to datasets with over 100,000 tips and provide a promising avenue for future versions of SciPhy.

We have adapted the discussion as follows to better reflect this:

While SciPhy offers promising avenues, it has some limitations. Tree space grows exponentially with the number of cells and current state-of-the-art −Bayesian inference methods may not converge for trees with more than around 1000 tips. Even for datasets of this size, ~~Bayesian phylogenetic~~ inference requires repeatedly evaluating the likelihood ~~in the range of billions of iterations~~ to reach convergence. Thus, despite the likelihood calculation already scaling linear with the number of tips, further speed-ups of the likelihood calculation can bring large benefits. We have achieved considerable improvements on the runtime of individual computations of the likelihood with caching and multi-thread parallelisation (and provide estimates of the resulting combined, overall runtime for convergence of typical analyses in Appendix Fig. 1). However, ~~though~~ the empirical data analysis on 1000 cells still required ~15 days, and would benefit from a further reduction in run-time. For future improvements, we aim to ~~further~~ accelerate the likelihood calculation using software optimizations or approximations, and expedite convergence on large datasets by confining tree space and adapting recent advancements based on mutation annotated trees [Sabeti et al. 2025] that have been demonstrated to scale to 100'000 tip trees.

5. Additionally, the authors mentioned the convergence issue of the SciPhy method. The authors should show the convergence statistics for each parameter in each dataset in the study, such as rhat and/or other statistics. For this issue, non-convergence of large datasets might be due to the high correlation between parameters, and the Differential-Evolution MCMC method (arXiv:1710.09486) might solve this problem.

We are glad to see this issue recognized and appreciate the crucial importance of clearly describing metrics that are employed to assess convergence. Throughout this study, we have relied on the Effective Sample Size (ESS) as convergence metric for all datasets. Specifically, we use a threshold of ESS value of 200 for all parameters of interest, which we now more explicitly mention in the main text. For all analyses of the HEK293T and gastruloid datasets, we now additionally report their values for the posterior in Appendix table 2, along with the rhat value as suggested. All analyses reported fulfill ESS values above 200 and rhat

values close to 1 and below 1.1, suggesting appropriate convergence properties. While its application to improve runtime in SciPhy is beyond the scope of our study (presented in real parameter space only) we also take good note of the potential of the suggested Differential-Evolution MCMC method (and mention this suggestion in Discussion).

Convergence metrics

Analysis	Posterior $\hat{r}$	Posterior ESS
Figure 3 - HEK293T culture	1.077371	1447
Figure 4 - HEK293T SciPhy	id. Fig. 3	id. Fig. 3
Figure 4 - HEK293T UPGMA ordered	1.002006	6141
Figure 4 - HEK293T UPGMA ordered + root scaling	0.9983593	449
Figure 4 - HEK293T UPGMA ordered + SciPhy scaling	1.004213	297
Figure 5 - Gastruloid	1.014567	833

Appendix 0—table 2. $\hat{r}$ and ESS convergence metrics for all analyses reported

The methods section was adapted to clarify this and refer to this table as follows:

For each subset of the data, we run 3 MCMC chains for at most 10^9 iterations. We assess convergence
by confirming that the three chains are sampling the same stationary distribution, with an ESS value per chain > 200 for all parameters of interest and visually in Tracer (Bouckaert et al. (2014)). We discard 10% burn-in and combine all 3 chains using LogCombiner (Bouckaert et al. (2014)). The combined ESS and rhat values for the posterior probability of all analyses of this dataset are reported in Appendix table 2.

The inference was performed as follows: we ran 3 MCMC chains on the dataset for at least 10^9 iterations or until convergence. We assessed convergence, and combined all 3 chains as previously described (see above). The combined ESS and rhat values for the posterior probability for our analysis of this dataset are reported in Appendix table 2.

The discussion was appended with suggested sampling approaches as shown below:

Improvements to other components of Bayesian inference, such as, e.g., MCMC sampling methods, may also provide additional runtime reduction but their applicability beyond continuous/real parameters contexts, such as the one encountered in phylogenetic inference, remains an open research problem.

Minor Comments:
In Figure 4a, it is unclear what the four coordinates for the RF distance comparison represent.

Thank you very much for pointing out this lack of clarity. Where mentioned, we have clarified that these are a result of dimensionality reduction using principal component analysis. We also emphasized that these dimensions do not represent interpretable features of the trees compared in the methods section.

E.g., amongst others: *For each metric, we perform multidimensional scaling to map these distances in lower dimensions using principal coordinate analysis (Gower (1966)).. For all mappings we find that 4 or more dimensions provide acceptable levels of distortion (trustworthiness x continuity > 8.5). These first 4 dimensions, which we label 1 to 4 in our plots, are used for reference but do not represent interpretable features of analyzed trees.*

The probability transition model in section 4.2 is the core part of the SciPhy model. However, this section is not very clearly written, with confusing symbol usage in eqn(1)-eqn(3) and a lack of detailed Explanations.

We are grateful for the attention to detail in this section. We highlight the revisions we made below and hope this makes the section more accessible:

1) We noticed that some instances of |c|, denoting the number of inserts, were not formatted correctly and appeared as —c— in the pdf. We fixed the formatting for all occurrences.

2) We clarified the two key steps for computing the transition probabilities:

We denote the transition probability between states a and b under the model introduced above as $P_{a,b}(\Delta t; \eta, r) = P(Y_{t+\Delta t} = b; Y_t = a, \eta, r)$*. In the following, we derive this transition probability. In step 1, we calculate the probability that a number of ~~of introducing any~~ |c| new edits have been inserted. In step 2, we calculate the probability that those edits correspond exactly to the tuple ~~of introducing the particular inserts in~~ c.*

3) We introduced various edits to improve clarity:

We calculate the probability of this event by targeting its complement, namely the probability that less than |c| ~~of any fewer~~ inserts ~~being~~ were introduced.

Let us assume that the |c| insertions having occurred from a to b are exactly the tuple $c = (s_i)_{i=|a|+1,\ldots,|b|}, s_i \in \Gamma$.
~~The probability of inserting c conditional on $|c|$ is the product of the individual insertion probabilities of all elements within the tuple c~~ Conditional on observing exactly |c| inserts, the probability of realising the specific tuple c equals the product over the ~~individual~~ insertion probabilities f_l *for each element of c (by independence, see Assumption 2b).*

$$P(c; |c| = j, \eta) = \prod_{k=|a|+1}^{|a|+j} P(s_k) = \prod_{k=|a|+1}^{|a|+j} \prod_{l=1}^{M} f_l^{\mathbf{1}(\gamma_l, s_k)}$$

Note that the indicator function $\mathbf{1}(\gamma_l, s_k)$ *serves as a one-hot selector, keeping the insertion probability* f_l *only when the edit* γ_l *matches the observed symbol* s_k *in the tuple c. This*

construction collapses the inner product to the single insertion probability corresponding to the realised edit at position k.

Reviewer #2 (Remarks to the Author):

The authors developed a mechanistic model of sequential genome editing, SciPhy, within the BEAST 2 framework, allowing for estimation of time-scaled phylogenies and cell population parameters.
SciPhy was validated using simulations and applied to two real datasets:

a) A monoclonal culture of HEK293T cells, where SciPhy estimated editing dynamics, cell lineage trees, and population growth rates over a 25-day period.

b) Murine gastruloids modeling early mammalian development, in which SciPhy analyzed complex growth dynamics during gastruloid formation, including changes in growth rates following CHIR treatment. Overall, the model formulation and inference appeared to be solid. However, there remains unanswered questions from the manuscript in terms of its application to real datasets. We raise the following major issues:

1. The most important result for time-scaled cell phylogeny inference is the cell tree. Because applications to the experimental data have no ground truth, the validation of the methodology and its accuracy under different circumstances is highly dependent on the simulation study. However, the simulation study presented here is inadequate. The author did not provide any results regarding the cell tree accuracies in the simulation study, only the model parameters were compared. It is necessary to compare the performance in both topology distance and branch length error. We suggest the following:

   a. The proposed method should be compared to UPGMA in terms of tree reconstruction accuracy under different parameters.

   b. The performance trend should be explored with respect to different editing parameters.

The results are necessary for evaluating the number of errors and advancement there is in terms of reconstruction accuracy.

We are thankful for such detailed suggestions on ways to improve this aspect of our study. Towards this goal, we have considerably extended our simulation study to evaluate the accuracy of cell trees inferred with SciPhy. For each of 100 simulated datasets used to validate SciPhy, we now systematically quantify the distance between ground truth simulated and inferred cell trees, covering both aspects of topology and branch lengths by using appropriate tree distance metrics (respectively, the Phylogenetic Information (PI) metric for and weighted Robison Foulds (wRF) metrics to account for branch lengths). This comparison to the ground truth simulated trees is additionally repeated for trees reconstructed using UPGMA and to random trees that serve as a null-hypothesis for the

ability to extract phylogenetic relationships from the data. Using this approach, we believe that each of your suggestions was addressed as follows:

a. “The proposed method should be compared to UPGMA in terms of tree reconstruction accuracy under different parameters.”
SciPhy outperforms UPGMA under all parameter settings explored, which we now show in Fig. 4, and Appendix Fig. 2. The difference is most pronounced under regimes of low editing rates and when factoring in branch lengths in addition to topology, which can be attributed to the additional information extracted by SciPhy, through its modelling of the edit probabilities. Please refer to the response to point 2 of Reviewer 1 for more detail on the benchmarking to UPGMA, and see Appendix Fig. 2 added below for dependency on editing parameters.

b. “The performance trend should be explored with respect to different editing parameters.”
The trend highlighted above is qualitatively robust to all explored editing parameter regimes. In relative terms (across different methods), as well as in absolute terms (for SciPhy alone), the editing rate has the largest effect on the resulting accuracy of inferred trees. For SciPhy alone, we show trends in reconstruction accuracy against simulation values of the editing rate, collision probability (the sum of squared edit probabilities, summarizing the skew in edit probabilities), and tree size, in Appendix figure 3 (see below).

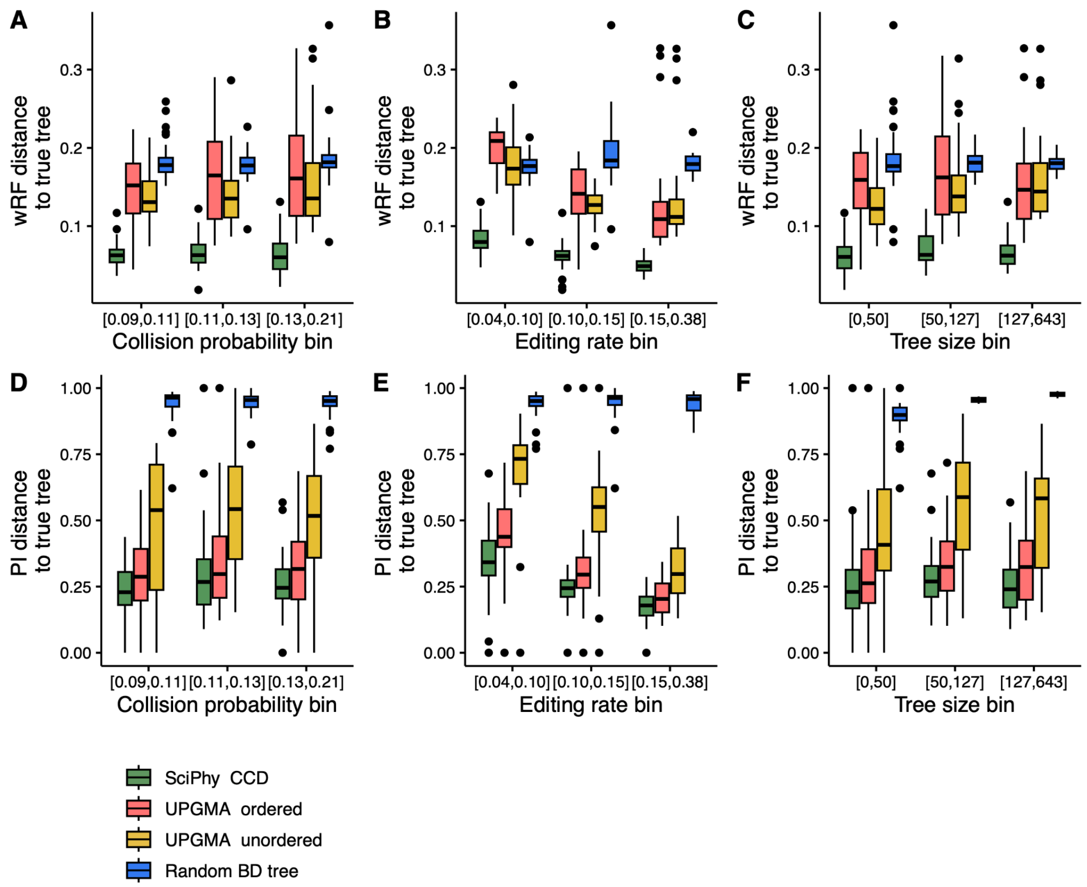

Appendix Figure 2: *Accuracy of SciPhy reconstructed trees w.r.t. different simulated parameter regimes, benchmarked to UPGMA on all validation datasets. For all datasets simulated in the validation study, we showcase the distance from the true tree to trees reconstructed with SciPhy (summarised using the Conditional Clade Distributions, denoted as SciPhy CCD), an order-aware UPGMA method (UPGMA ordered), the standard UPGMA method, ignoring the order between edits (UPGMA unordered), and randomly generated through simulation under the birth-death sampling model (Random BD tree), where UPGMA trees are all scaled to 25 days for comparison. These distances are shown for binned simulation parameters values and calculated using the weighted Robinson Foulds (wRF, panels A-C) metric and Phylogenetic Information metric (PI, panels D-F) The "collision probability" presented here is the sum of squared insert probabilities, and represents how skewed towards certain inserts the editing process is.*

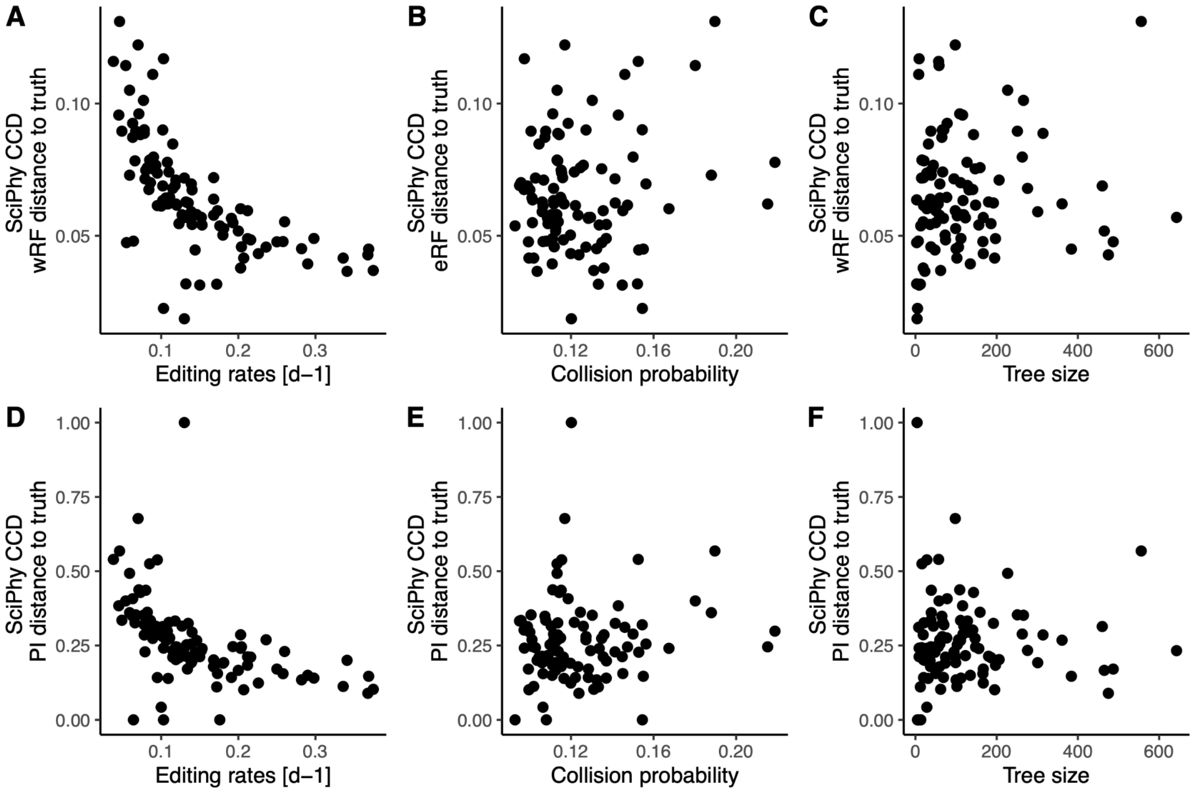

Appendix figure 3: *Accuracy of SciPhy reconstructed trees w.r.t. simulated parameter values on all validation datasets. For all datasets simulated in the validation study, we showcase the distance from the true tree to trees reconstructed with SciPhy (summarized using the Conditional Clade Distributions, SciPhy CCD). These distances are shown against simulation parameters values and calculated using the weighted Robinson Foulds (wRF, panels A-C) metric and Phylogenetic Information metric (PI, panels D-F). The "collision probability" presented here is the sum of squared insert probabilities, and represents how skewed towards certain inserts the editing process is.*

2. One major concern in Bayesian inference applied to cell lineage tracing data is scalability, especially the performance with many cells. We think a scalability analysis is necessary. The author should apply the methods to simulated datasets of varying number of cells and sites, and quantify trend of runtime, as well as the performance in terms tree reconstruction error at different running time. Regarding this point, the author has made qualitative statements

such as “MCMC methods searching tree space may not converge for trees with more than around 1000 tips”. A quantitative analysis of the scalability of the method is necessary, including the conditions under which the method will not converge, etc.

Thank you for this valuable comment. We’ve expanded our previous, qualitative assessment of SciPhy’s scalability with quantitative estimates of the runtime until convergence, assessed on the 100 simulated datasets from our validation study, which range from minutes for smaller datasets to days in the largest datasets, approaching 1000 cells. This is now reported in Appendix figure 1 (see below). From this, corresponding trends w.r.t. varying sites can be coarsely estimated, as individual evaluations of the likelihood scale quadratically with the number of sites. The performance in terms of tree reconstruction error is also now systematically reported for all of the simulated datasets within our simulation study, as shown above.

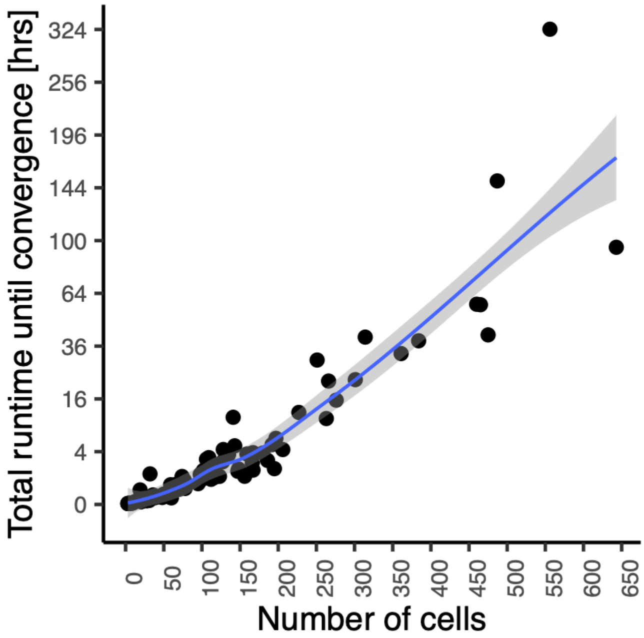

Appendix figure 1: Approximate runtime for convergence of a SciPhy analysis We report the runtime required for convergence of all datasets run in the validation study. We show here the recorded, empirical total runtime rescaled to reach a minimum ESS value of 200 for the SciPhy likelihood.

This figure is explicitly referred to in discussion as follows:
We have achieved considerable improvements on the runtime of individual computations of the likelihood with caching and multi-thread parallelisation (and provide estimates of the resulting combined, overall runtime for convergence of typical analyses in Appendix figure 1).

The methods section describing the implementation and the simulation study both refers to these runtime trends:

- Implementation subsection:

Additional speed-up is achieved by computing the likelihood for each tape alignment in parallel, which is easily achieved with existing components in BEAST 2 that support threading. These software optimizations lead to an overall 8-fold speed up relative to the naive implementation for an analysis involving 1000 sequences using 6 computing cores on the "Euler" scientific computing cluster. Using this setup, realistic estimates of the average runtime required for convergence on smaller datasets from the validation study are provided for reference in Appendix figure 1.

- Validation subsection:

Lastly, we calculated the coverage as the proportion of times the simulated (true) value fell within the estimated 95% HPD (Highest Posterior Density) interval. For each of these datasets, we estimate the total runtime necessary for convergence by weighting the total empirical runtime by the ratio between the final obtained ESS and ESS 200 Lastly, we calculated the coverage as the proportion of times the simulated (true) value fell within the estimated 95\% HPD (Highest Posterior Density) interval. For each of these datasets, we estimate the total runtime necessary for convergence by weighting the total empirical runtime by the ratio between the final obtained ESS and the desired minimum ESS of 200, as reported in Appendix figure 1.

3. Adding to the last two points, the simulation study can and should also be used to evaluate the exact experimental scenarios in which the method was applied later in the manuscript to offer an idea of the amount of error in this estimation. For example, what is the average error in the inferred time of divergence in both experiments?

We are grateful that this important consideration was raised. Indeed, our validation study was designed to mimic experimental scenarios inspired from the empirical dataset analysed later in the manuscript, without losing generalisable properties for the application of SciPhy to other/upcoming, subsequent datasets. For this, we chose a validation setup where simulated barcodes have the same number of sites as both the HEK293T and gastruloid datasets (with array lengths of 5 sites), and where each cell harnessed 10 copies of the lineage tracing barcode (the HEK293T cell and gastruloids dataset contained 13 and 8 copies, respectively. Using these barcode designs, simulations were performed along a division process of at most 25 days, also covering realistic experimental lengths. Following their stochastic nature, our simulations cover a range of editing rates and probabilities which we believe provide sufficiently realistic intuition on SciPhy's behaviour. Using these as input we now also quantify the accuracy in branch length (divergence time) estimation with the weighted Robinson Foulds distance to the true tree. The results of this quantification, as well as corresponding changes to the manuscript, can be found in response to your first point above as well as to Reviewer 1's second point. Related points on examining the tree reconstruction problem with respect to different experimental designs are also mentioned in our discussion, as discussed further below.

Furthermore, more generalizable/theoretical statements on the expected information content and potential accuracy of reconstructed trees using a wide range of experimental scenarios is also available in related work from colleagues. This is now explicitly mentioned in our discussion. Please refer to the response to your suggestion below, on comparing SciPhy to "traditional recorder without sequential information"

4. In the HEK293T analysis, the author suggested that the early and late edits may have different rates, but it is unclear if it can be concluded that there is no difference by limiting the data to just the first two edits since the model is mis-specified. The robustness of the estimates under the scenario where early and late edits have different rates can again be tested by simulating data where the early edits are faster and applying the wrong model. One solution is to relax the uniform editing rate assumption for early and late estimates.

We thank the reviewer for this suggestion. We agree that relaxing the assumption of identical editing rates across sequential sites would be a more direct approach to testing for the presence of early or late sites being edited at different rates. However, incorporating site-specific rates into the SciPhy likelihood presents substantial technical challenges.

In the current model, edits proceed sequentially along a tape and occur according to a homogeneous Poisson process, such that the probability of observing kkk edits within a given time interval has a closed-form expression. If editing rates instead vary by site, the editing process becomes a nonhomogeneous poisson process where the rate changes occur after the previous editing event has happened. Computing the probability of k edits under such a process has no closed form solution and requires integration over all possible editing trajectories. This renders direct likelihood computation intractable and would require substantial changes to the inference strategy. We agree that future work could explore such an extension.

To address the reviewer's concern within the current framework, we performed a simulation as suggested and added Supp. Fig. 6. Specifically, we adapted the simulation class to allow for varying editing rates across sites and then applied the mis-specified model as done before. We simulated data for 12 tapes, where tapes 1-9 have 5 sites and tapes 10-12 have 4 sites (highlighted in blue in figure below). In scenario one (top row in figure - “No rate diff”), all sites have the same edit rate. In scenario two (bottom row in figure - “Rate diff”), site 5 is edited at 20% of the original rate. Thus, in this scenario the 4-site tapes do not have that site that is edited slower.

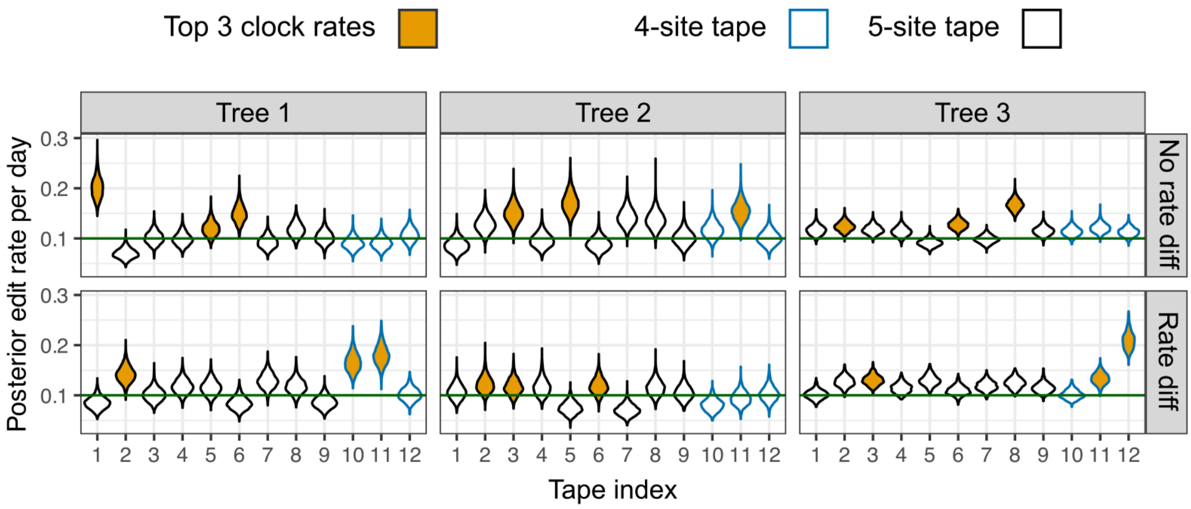

Supp. Fig. 6 Effect of within-tape rate variation on inferred editing rates. Posterior estimates of edit rates per tape under SciPhy for three simulated trees. In each panel, tapes are shown along the x-axis (tape indices 1–12), with violin plots representing the posterior distribution of

the per-tape edit rate. Tapes 10–12 (blue outlines) are 4-site tapes; others are 5-site tapes. Orange-filled violins indicate the three tapes with the highest median inferred edit rates. Top row: scenario with no rate differences between sites. Bottom row: scenario where the 5th site is edited at 20% of the base rate. In the "Rate diff" condition, 4-site tapes more frequently appear among the fastest edited rates, as they lack the slower 5th site.

We expect that SciPhy when used as a mis-specified model will estimate an average edit rate across the sites. Thus, given that the 4-site tapes do not contain the slowly edited site, we expect their estimated edit rate to be on average higher than their 5-site counterparts in scenario 2. Indeed, in scenario 2, the 4-site tapes are more often among the 3 highest clock rates (highlighted in orange, 4/9 vs 1/9).

Thus our simulations provide some preliminary evidence that rate variations between sites within a tape leave a trace in the edit rate per tape estimated by SciPhy even if it is misspecified. However, we here only tested a single rate variation and more complex variations in edit rates would surely need an extension of the likelihood approach compared to the simple statistical test we used before. Further, given the fluctuations in edit rate estimates in scenario 1, when no rate difference is present, we soften our conclusions and conclude that interpreting the edit rates is more meaningful when multiple replicates are present.

Therefore, we adapted the results section as follow:

We then compared the 4x and the 5x tapes. In the analysis using all sites, the three highest editing rates among the 4x and 5x tapes were all 4x tapes. This result is surprising and statistically significant under the assumption of equal rate (p(k=3)=0.005, see Methods for test details). However, in the analysis using only 2 sites, this order was lost, with only one 4x tape among the three tapes with fastest editing rates. This result was not statistically significant under the assumption of equal rates (p(k=1)=0.5). Therefore, given the editing rate estimates under SciPhy, the difference in editing rate between 4x tapes and 5x tapes may be due to a difference in editing speed across the last 2-3 sites, for instance slower editing at the 5th site of the 5x tapes. To further explore this scenario, we simulated data in which the 5th site was edited at 20\% of the original rate. In this scenario -but not under equal edit rates-we observed two 4x tapes being among the 3 tapes with the highest inferred editing rates (see Appendix Fig. 3), supporting our earlier statistical analysis. However, as our HEK293T analysis is based on a single experimental replicate, additional replicates would be required to draw definitive conclusions on the relative edit rates.

We also now more explicitly acknowledge this limitation in the revised discussion:

In our analysis of cell culture growth, we observed that the inferred editing rates for the 4-site tapes were consistently higher than those for the 5-site tapes. We used a statistical test to show this pattern is unlikely to arise under a null model of equal rates across all tapes. However, a limitation of our approach is that all inferences were performed using SciPhy, which assumes equal editing rates across sites. We present simulations that provide evidence for SciPhy estimating an average edit rate across sites for each tape, essentially allowing us to constrain the space of within-tape rate variation to scenarios consistent with

the estimated per-tape average. Nevertheless, a model that explicitly allows for site-specific editing rates would enable more direct testing of such hypotheses and remains an important direction for future work.

We think this work can also be much improved by addressing in addition the suggestions below:

1. One natural question regarding the lineage tracing using a sequential recorder is how this system compares to a more traditional recorder without sequential information. As the authors have also previously done work on traditional recorders. It will be natural and highly valuable to provide a comparison of the two under comparable number of recording sites and rates.

This is a very relevant consideration. As suggested, we have now extended the manuscript with a direct comparison to traditional recorders without sequential information on simulated data with the same sites and rate, providing intuition into the differences between these technologies, using UPGMA and TideTree (for full details, please see our response to point 2 of Reviewer 1 . Further simulation based studies, as well as theoretical results on the information content of sequential recorders have also provided more insight into these differences and are now referred to in our discussion.

As a mechanistic model, SciPhy can directly account for these factors and even use the signal from tapes being edited at different rates to inform branch length estimation. This ability to estimate branch lengths, and from them, cell population dynamics is consistent with a growing body of related work assessing properties of sequential editing data Mulberry and Stadler (2025); Pilarski et al. (2025).

2. The implementation of the model is heavily reliant on the BEAST2 framework, but the details on how this model is implemented within BEAST2 are not very clearly described. As people in cell biology may not be familiar with the framework, some details should be presented that are approachable by someone unfamiliar with BEAST2. For example, which components of BEAST2 have been modified in which way.

We agree that further clarification of our BEAST 2 implementation will make the manuscript more accessible to readers less familiar with the framework. In response, we have revised the relevant paragraph in the Methods section to outline which components of BEAST 2 were extended or replaced.

We implement the above editing model alongside an edit sequence simulator based on the feast package Vaughan (2024b) and a likelihood calculation ~~in JAVA~~ as a new package –SciPhy– for the ~~software~~ phylogenetic inference platform BEAST 2. Specifically, we introduce a new substitution model class, SciPhySubstitutionModel, which replaces standard models such as Jukes–Cantor to compute transition probabilities between edit states. We also extend the Generic TreeLikelihood class in BEAST 2 with the SciPhyTreeLikelihood class tailored to sequential editing. This implementation leverages domain-specific properties of lineage tracing data—for example, that the set of possible ancestral sequences

is known. Upon installing the SciPhy package, these components are directly available to BEAST 2 users via both the command-line interface and the graphical interface BEAUti.

3. The branch length estimates are expected to be better if more accurate prior information on editing rates is available. Similarly, tree inference should improve given prior estimates on insert probabilities. Generally, these parameters are not hard to measure experimentally for a given system. The author can demonstrate the effect of having prior on these and the improvements that can be made in simulation.

This is a very relevant consideration in the context of the artificial genetic constructs that SciPhy is designed to deal with, where smaller, control experiments or replicates may be run to provide more confident measurements of the editing rates and insertion probabilities. This consideration is also valid for all related lineage tracing technologies. However, the majority of lineage tracing datasets available having consisted so far of single replicates, obtaining in practice such precise priors remains a challenge. Likewise, controlled, small scale experiments may not provide sufficient insight into the time and context dependent behaviour of lineage tracing constructs. For these reasons, we remain cautious of providing overly confident priors in the context of this study, and use relatively broad (but experimentally informed) priors throughout.

Nevertheless, towards that point, we have assessed the effect of prior choices in our analyses of the HEK293T and gastruloid dataset, and found that parameter inference is robust to various prior settings, including misspecified ones. Further discussion on testing the sensitivity of our results to these, as well as more details on the choice and availability of prior experimental information can be found in response to the third point raised by Reviewer 3.

Reviewer #3 (Remarks to the Author):

Seidel et al. introduce SciPhy, a Bayesian phylogenetic framework for reconstructing time-scaled lineage trees and inferring cell population dynamics using CRISPR-based sequential genetic lineage tracing data. They integrate the framework into the BEAST 2 platform, enabling the analysis of sequential genome editing data that tracks not only genetic edits but also the order of their accumulation. SciPhy was validated through simulations and applied to real-world datasets, including lineage tracing data from HEK293T cells and murine gastruloids. Compared to traditional clustering methods like UPGMA, SciPhy demonstrates improved accuracy in tree topology and allows for the simultaneous inference of parameters such as cell division rates, editing rates, and sampling proportions. Specific strengths include its capacity to handle uncertainty in lineage relationships and its ability to estimate time-resolved growth dynamics, which is particularly advantageous for datasets with dynamic lineage tracing.

The manuscript was well written and here are some suggestions that may enhance the manuscript:

* The manuscript does not provide sufficient discussion on how SciPhy handles missing or noisy lineage tracing data, which is common in CRISPR-based lineage tracing due to

dropout, sequencing errors, or incomplete editing. Missing data can skew tree reconstruction, particularly when certain edits or cells are underrepresented, leading to biased estimates of population parameters. The authors need to implement a model that explicitly accounts for dropout and sequencing errors, as seen in other tools like the PMM model in Mai et al. 2024. The authors need to add simulation studies to assess SciPhy's robustness under varying levels of data noise and missingness. The authors should also include a discussion on limitations due to missing data and the effect on parameter inferences.

We thank the reviewer for this comment, and acknowledge that dropout and error modeling is currently a gap in this study. We have expanded discussion on this topic, referring to the suggested work by Mai et al., 2024 as well as related work by Chen et al.,2022. We now make mention of the fact that datasets analysed in our study also suffer from such loss, where incomplete alignments make up a significant portion of sampled cells. In SciPhy, accommodating such lossy data may be achieved by modifying the likelihood formulation to account for states representing the effect of e.g., heritable, time-dependent, silencing as well as non-heritable sources of loss, such as sequencing dropout. This extension of SciPhy, along with extensive simulation studies to assess the effect of varying levels of missing data under different parameter regimes, is currently ongoing work that we envision to release in parallel with the present manuscript. An initial, validated, version of this extension is already publicly available to SciPhy users.

The discussion was updated as follows:

~~A common~~ Another limitation ~~in~~ shared by all CRISPR-based barcoding technologies is that resulting lineage tracing datasets are typically lossy as a result of the sparseness of single cell RNA-seq and/or silencing of the regulatory elements that drive transcription of recording substrates (e.g. DNA Tapes). Jointly, these mechanisms result in the partial recovery of ~~where not all~~ DNA Tapes per cell ~~are recovered in every cell - a problem that SciPhy does not currently model. This dropout is a consequence of the technologies used to readout lineage recordings, such as single-cell RNA-seq, that result in loss of tape sequences for some cells.~~. For example, in the HEK293T cell culture dataset used in our study, none of the tapes were recovered for all sequenced cells, and there was variability in the rate of recovery of individual tapes. The relative contribution of either mechanism to this loss is unknown and for ordered lineage tracing data, and ~~current~~ state-of-the art lineage reconstruction approaches ~~often~~ have thus far neglected cells with incomplete sets of DNA Tapes ~~-resulting in data loss~~. Ignoring such sources of loss may bias tree or population parameter inference, e.g. by violating a common assumption of birth-death models of uniform sampling of the population of interest. Related work for single-cell diploid data and CRISPR-based unordered data has shown that mechanistic models of such error sources improve features of inferred lineage trees (Chen et al., 2022, Mai et al.,2024), especially relating to branch lengths . ~~Future~~ Follow up work should integrate a dropout model accommodating tape absence such that data loss and the resulting bias is avoided.

Bayesian inference in SciPhy involves computationally intensive MCMC methods, which may become infeasible for datasets with thousands of cells. This will severely limit SciPhy's application to larger lineage tracing datasets, particularly those involving whole-organism or long-term lineage studies. I would like to see the authors to explore parallelized or

approximate inference methods, such as variational inference or Bayesian posterior approximations, including benchmarks comparing SciPhy's performance on large datasets with other tools like UPGMA and GAPML.

We acknowledge that it is of crucial importance to consider the current limitations of SciPhy with respect to computational scalability. We have made a few revisions towards more clearly describing them to readers, while highlighting that the bottleneck in SciPhy's scalability is not in its current formulation (the SciPhy likelihood scales linearly with the number of analysed cells) but in the fundamental approach of Bayesian phylogenetic inference, where the analysis of any type of data will be limited by the ability to explore the space of tree topologies (which scales exponentially with the number of cells). To provide a better intuition of SciPhy's scalability on realistic datasets sizes, we now report estimates of the runtime required for the datasets used in our validation study, and mention promising directions that address the fundamental issue of scalability, with mutation annotated trees [Sabeti et al. 2025] with potential to reach scales of 100,000 tips in future versions of SciPhy. This is now primarily addressed in our discussion:

While SciPhy offers promising avenues, it has some limitations. Tree space grows exponentially with the number of cells and current state-of-the-art −Bayesian inference methods may not converge for trees with more than around 1000 tips. Even for datasets of this size, ~~Bayesian phylogenetic~~ inference requires repeatedly evaluating the likelihood ~~in the range of billions of iterations~~ to reach convergence. Thus, despite the likelihood calculation already scaling linear with the number of tips, further speed-ups of the likelihood calculation can bring large benefits. We have achieved considerable improvements on the runtime of individual computations of the likelihood with caching and multi-thread parallelisation (and provide estimates of the resulting combined, overall runtime for convergence of typical analyses in Appendix fig. 1). However, . However, ~~though~~ the empirical data analysis on 1000 cells still required ~15 days, and would benefit from a further reduction in run-time. For future improvements, we aim to ~~further~~ accelerate the likelihood calculation using software optimizations or approximations, and expedite convergence on large datasets by confining tree space and adapting recent advancements based on mutation annotated trees [Sabeti et al. 2025] that have been demonstrated to scale to 100'000 tip trees.

While the implementation of approaches that would enable the analysis of datasets of more than thousands of cells is beyond the scope of the current study, we nevertheless provide more thorough benchmarking as suggested. Our comparisons now include three phylogenetic inference methods, and assess their relative performance on both simulated and empirical data (most of the section "Comparison to state-of-the-art" has been updated with these results). Our comparisons do not include GAPML (and GABI, its Bayesian counterpart) as it specialises in dealing with barcode designs prone to large deletions, which are not a consideration in SciPhy data. Instead, we use TiDeTree from BEAST and UPGMA, providing a reference to analogous methods dealing with unordered barcode editing. Figure 4 and Appendix figure 2 both highlight that SciPhy generally outperforms all these alternative methods. For more discussion of these aspects, please also refer to our responses to Reviewer 1's 2nd and 3rd points.

* The dependence on priors for editing rates, insertion probabilities, and population parameters was not thoroughly validated against empirical data. Over-reliance on priors may bias results, particularly if the priors are poorly chosen or do not reflect experimental realities. I would like to see sensitivity analyses to evaluate the impact of different prior distributions and use empirical datasets to justify the choice of priors and refine them accordingly.

We agree that careful assessment of prior assumptions is critical, particularly in settings where empirical calibration is limited. In the revised manuscript, we now include additional sensitivity analyses evaluating the robustness of our results to alternative prior distributions on the editing rates and insertion probabilities (see Supplementary Figs. 15 and 16).

We did not vary the prior distributions for the population growth rate, as these priors were already broad, and we explored alternative phylodynamic models in the main text. Across these models, the key features of the inferred dynamics remained consistent.

Focusing on the editing model parameters, we tested a range of broad, informative and even intentionally misspecified informative priors (e.g., a Gamma(2, 0.1) prior on the edit rate). In all cases, the qualitative and quantitative features of the inferred editing and population dynamics were remarkably robust.

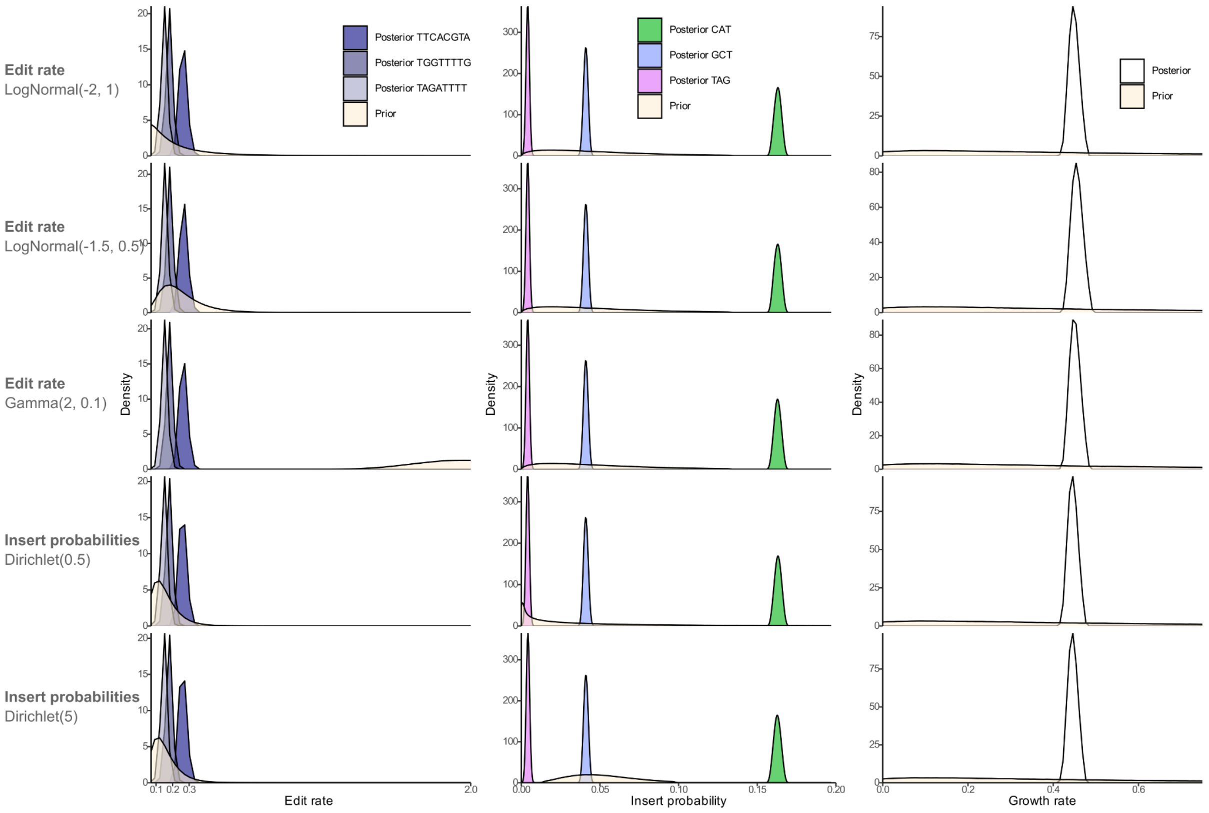

Supp. Figure 15. Results of the phylodynamic analysis of cell culture growth are robust to different choices of prior distributions.

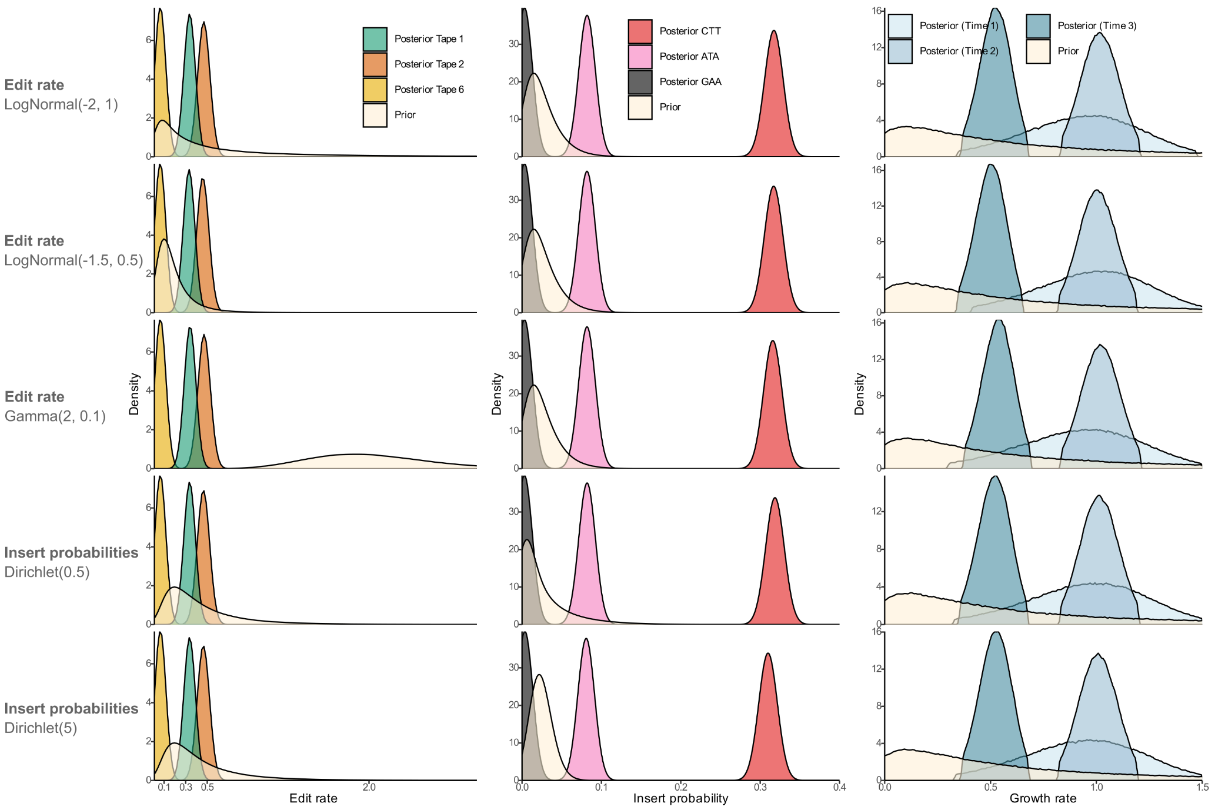

Supp. Figure 16. Results of the phylodynamic analysis of gastruloid growth are robust to different choices of prior distributions.

Furthermore, while direct empirical measurements of editing dynamics were not available for the specific experimental setups analyzed, our priors were designed to span a wide range of biologically plausible scenarios, informed by prior experimental reports on related editing systems. We now clarify this rationale and explicitly cite supporting studies in the Methods section:

The updated Methods section describing the cell culture analysis now reads as follows:

We place prior distributions on the parameters of the editing model as described in table \ref{tab:inference-priors}. As no quantitative information on the editing dynamics is available for this experimental setup, we use broad priors that capture a variety of experimentally plausible values (as in the validation part). For example, the clock rate prior spans between 1 and 10 edits per tape over 25 days. As each tape has a maximum of 5 sites, 10 edits correspond to the scenario of early saturation. The prior is centered on 5, the desired edit count. The prior on the edit probabilities is a Dirichlet prior with concentration 1.5, chosen to reflect expected asymmetries in editing outcomes observed in similar non-sequential recorders Gong et al. (2021).

[...]

We set priors on the birth and death rate (Tab. 4) that are very broad; leading for instance to between 12 and $2.5 * 10^{30}$ *cells at the time of sequencing, which broadly contains the reported number of cell (*$1.2 * 10^{6}$*).*

And the updated Methods section describing the gastruloid analysis now reads as follows:

We use SciPhy to model the prime editing process throughout the experiment for the DNA Typewriter tapes, where, as previously, each of the 8 tapes acquires edits at an independent rate, but where insertion probabilities for the 42 trinucleotide characters are shared across the tapes. <u>We use the same prior distribution on the insertion probabilities as in the cell culture analysis, based on the expectation that editing outcomes are not uniformly distributed. For the clock rate, we adjust the prior to reflect the shorter 11-day time frame of the gastruloid experiment. The 95% highest density interval again corresponds to an expected range of 1 to 10 edits per tape, with the median aligned with the desired outcome of 5 edits</u> (see Table 5 for prior distributions).

We believe these additions address the concern about potential over-reliance on priors.

POINT BY POINT RESPONSE

Reviewer comments are reproduced in full below, with our responses provided inline in **blue**. Text quotes from the edited manuscript are *italicised*, new text is underlined, deleted text is ~~struck-through.~~

REVIEWER COMMENTS

Reviewer #2 (Remarks to the Author)

The authors have addressed many concerns and comments from the initial review, including more simulation validation, benchmarking against alternative inference methods, and improved clarity of model and BEAST2 implementation. These updates strengthen the paper. However, two important points remain that would improve the completeness of the Manuscript:

1. While the authors provided a detailed runtime analysis as a function of the number of cells, scalability with respect to the number of editing sites remains untested. Understanding scaling performance with number of sites is valuable theoretically for the readers and important for guiding future applications. The author should test the scaling at intermediate cell counts and increasing site numbers.

Thank you for this comment. We appreciate the importance of adding analyses of how SciPhy scales with respect to the number of sites, as this is of high practical relevance to users. Following this suggestion, we now include an additional figure (Appendix figure 2) showing a linear dependency of the total runtime needed for convergence of an analysis as a function of the number of sites per tape, for reconstruction of one of the simulated trees from our validation study (containing 123 cells). In combination with our existing runtime analysis w.r.t. the number of cells (Appendix figure 1), this should provide a general intuition on the expected runtime for a wide variety of scenarios to readers. The new figure and its reference in the text are shown below:

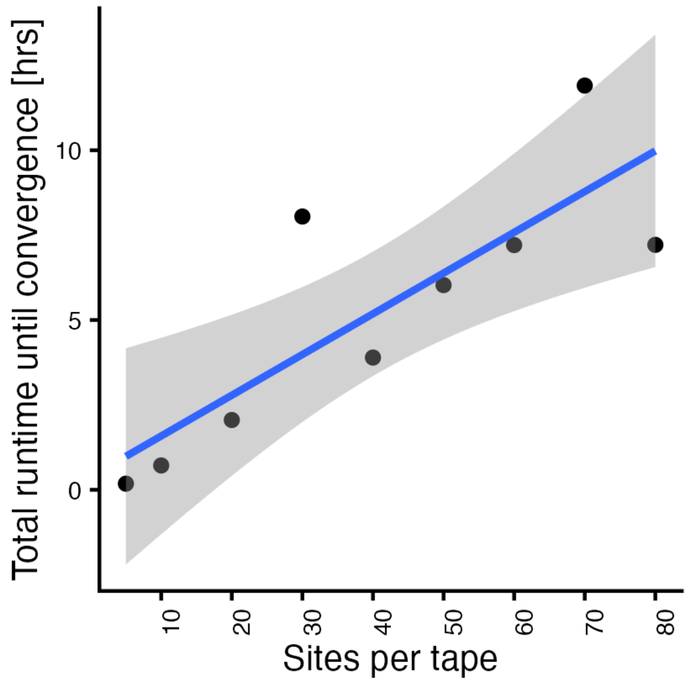

Appendix figure 2. Approximate runtime needed for convergence of a SciPhy analysis w.r.t. tape length. We report the runtime required for convergence of the lineage tree reconstruction and parameter estimation based on 123 cells harboring a single tape, varying the number of sites per tape. We display here the empirical total runtime rescaled to reach a minimum ESS value of 200 for the SciPhy likelihood.

In the revised Methods section:

“These software optimizations lead to an overall 8-fold speed up relative to the naive implementation for an analysis involving 1000 sequences using 6 computing cores on the “Euler” scientific computing cluster. Using this setup, we provide realistic estimates for the average runtime required for convergence on smaller datasets from the validation study, and when varying the number of editing sites in Appendix Fig. 1 and 2.”

2. We appreciate that a methodological extension for dropout modeling could be the subject of a separate manuscript. However, simulations incorporating dropout and sequencing noise fall within the scope of this study. The current model should be stress-tested under realistic experimental error scenarios (e.g., missing tapes, truncated barcodes, sequencing errors), particularly because these issues are relevant in the datasets analyzed. Including a simulation with different levels of noise is essential for this work.

We acknowledge that addressing the effect of different sources of noise and missing data is of relevance in this study. To cover this aspect, we have amended the Appendix with a simulation study that assesses the effect of 2 sources of missing data which we believe to be an issue for all currently available datasets: transgene silencing and sequencing dropout. Both sources of noise contribute to incomplete recovery of tapes per cell. We simulated 100 datasets, varying the amounts of tape loss arising through both processes, and now report tree reconstruction accuracy achieved with these incomplete datasets in terms of topology and branch length, using the PI and wRF metrics (as well as the impact on parameter inference). Changes made towards addressing this point are described below:

In the Results section:

Finally, we evaluate SciPhy's robustness to two major sources of missing data found in lineage tracing datasets, namely, transgene silencing and sequencing dropout. To do so, we simulate alignments under varying levels of heritable tape loss (modeling transgene silencing) and loss upon sampling (modeling sequencing dropout), which both result in incomplete recovery of tape sequences per cell (see Materials and Methods for a description of this simulation process). Trees reconstructed using incomplete barcode alignments are farther from the ground truth tree than those that use complete ones as input (see Appendix fig. 23, top and 24), considering both the tree topology alone (using the PI distance) and branch length estimation (using the weighted Robinson Foulds (wRF) distance). We find that the PI distance for tree topology is particularly sensitive to tape loss and the resulting alignment sparsity (see Appendix fig. 23 bottom left and 24, left), while the wRF metric considering both topology and branch lengths results in a weaker increase in distance as tape loss probability increases.

In the Methods section:

We assess the effect of alignment sparsity on the accuracy of phylogenetic reconstruction using SciPhy by generating incomplete alignments as input data for the same inference setup as used in the validation study. We consider two main mechanisms leading to tape loss: heritable loss of tapes at a constant rate through time, capturing e.g. transgene silencing, and tape dropout at sequencing, assuming that tapes will fail to be captured by the sequencing pipeline with a given probability. We simulated the editing process with loss for 20 independent tapes along each phylogenetic tree, and chose wide distributions for the rate of heritable loss and dropout probability, such that we would expect between 9.7 and 88% of sequenced tapes to be missing per simulated alignment (see Appendix Tab. 7). This led to an average of 11 tapes recovered per cell over all simulations (see Appendix Fig. 26). For each of these lossy tape alignments, we generate input alignments by constructing the largest set of tapes without missing data that include at least a third of all cells (“Filtered lossy data”). We used these alignments to infer the phylogenetic tree for the

subset of cells, as well as the editing and growth parameters, and assessed the accuracy in lineage reconstruction as described above.

In the Discussion:

Another limitation shared by all CRISPR-based barcoding technologies is that resulting lineage tracing datasets are typically lossy as a result of the sparseness of single cell RNA-seq and/or silencing of the regulatory elements that drive transcription of recording substrates (e.g. DNA Tapes). Jointly, these mechanisms result in the partial recovery of DNA Tapes per cell. For example, in the HEK293T cell culture dataset used in our study, none of the tapes were recovered for all sequenced cells, and there was variability in the rate of recovery of individual tapes. The relative contribution of either mechanism to this loss is unknown and for ordered lineage tracing data, state-of-the art lineage reconstruction approaches have thus far neglected cells with incomplete DNA Tapes. In our simulations, we found that trees reconstructed from incomplete alignments of DNA tapes are less accurate, but that parameters inferred jointly with those trees remain overall unbiased (see Appendix Fig. 25, for the editing and growth rates). However, ignoring such sources of loss may still bias tree or population parameter inference, by violating a common assumption of birth-death models of uniform sampling of the population of interest, e.g. if DNA tapes are simultaneously lost for groups of related cells. Related work for single-cell diploid data and CRISPR-based unordered data has shown that mechanistic models of such error sources improve features of inferred lineage trees Chen et al, 2022, Mai et al. 2024, especially relating to branch lengths. Follow up work should integrate a dropout model accommodating tape absence such that data loss and the resulting bias is avoided, and thoroughly investigate more complex patterns of loss.

Figures:

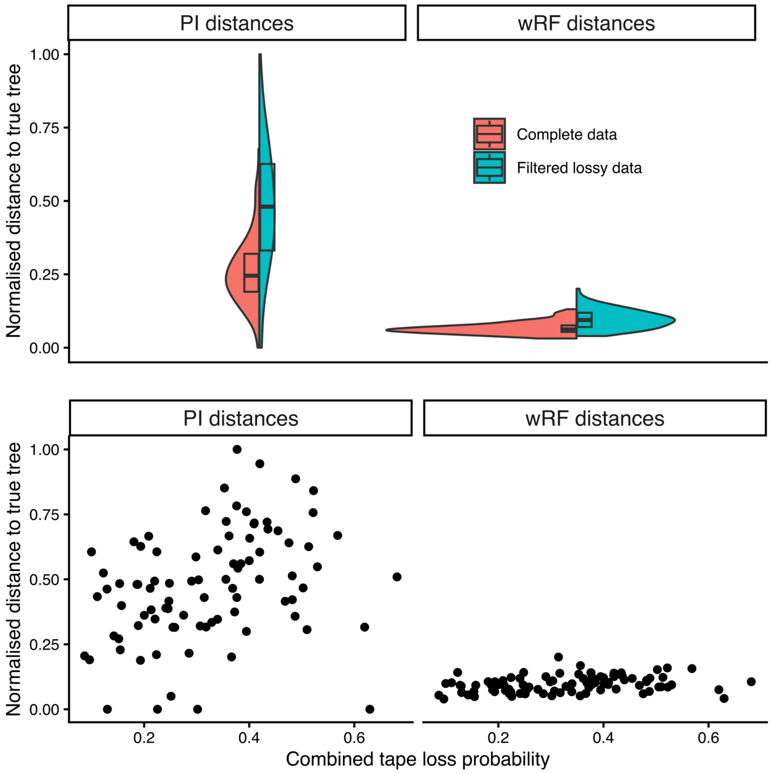

Figure 23. Robustness of tree inference using SciPhy to sparsity in tape alignments. In the top panel, for all trees simulated in the validation study, we showcase the distance from the true tree to trees reconstructed with SciPhy (summarized as point estimates with the conditional clade distribution (CCD) algorithm), using complete alignments of 10 tapes ("Complete data") or lossy alignments ("Filtered lossy data") of 20 tapes (leading to an average of 11 tapes after filtering) as input. In the bottom panel, we additionally show these distances against the combined tape loss used for simulation of lossy alignments.

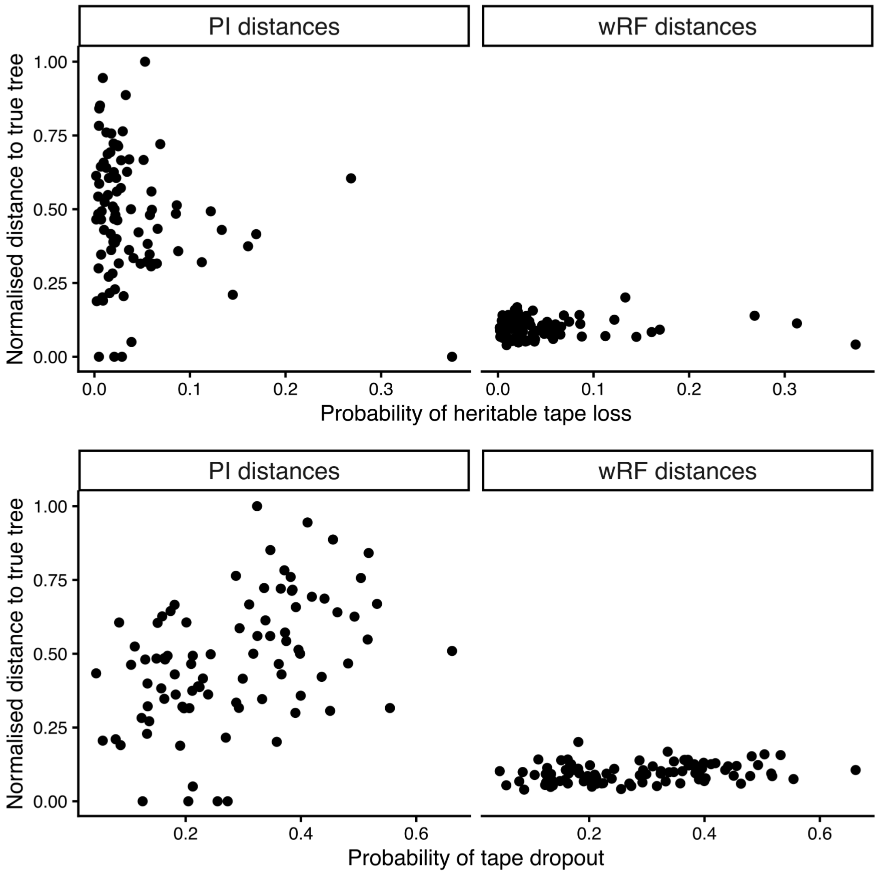

Figure 24. Robustness of tree inference using SciPhy to sparsity in tape alignments. For all trees simulated in the validation study, we showcase the distance from the true tree to trees reconstructed with SciPhy (summarized as point estimates with the CCD algorithm) using lossy alignments ("Filtered lossy data") of 20 tapes as input. In the top panel, we show these distances against the heritable tape loss probability (or transgene silencing probability) used for simulation, while in the bottom panel we show these distances against the tape dropout probabilities (or probability of loss upon sampling)

Parameter name	Symbol	Distribution	HDI
Clock rate	r	Log-normal ($\mu = -2, \sigma = 0.5$)	[0.03, 0.3]
Edit probabilities	f_k	Dirichlet ($\alpha = 1.5$)	[2e-4, 2e-1]
Heritable barcode loss rate	N/A	Log-normal ($\mu = -5, \sigma = 1.0$)	[0.00095, 0.048]
Dropout probability	N/A	Beta ($\alpha = 3, \beta = 7$)	[0.075, 0.6]

Table 7. Distributions for the editing model parameters used to simulate incomplete barcode alignments

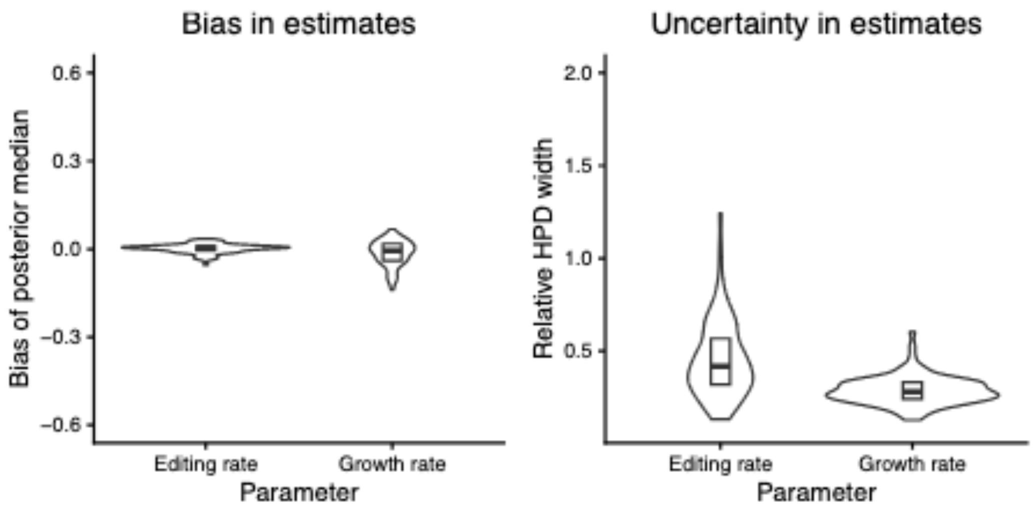

Figure 25. Robustness of parameter inference using SciPhy to tape silencing and dropout. For all datasets simulated in the validation study, we showcase the bias (left) and uncertainty (right) in editing and growth estimates obtained using lossy alignments ("Filtered lossy data" in Figure 23) of 20 tapes as input. The relative HPD width reported here is the width of the 95\% HPD interval divided by the true parameter value.

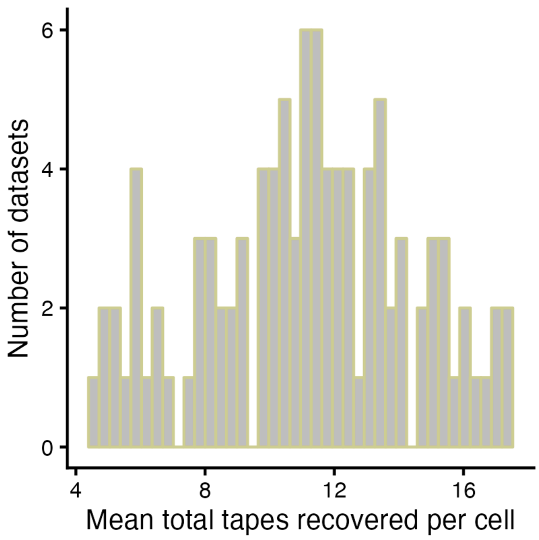

Figure 26. Mean number of tapes recovered per cell per dataset for the simulations of incomplete alignments, out of a total of 20 original tapes.

We also appreciate that tape truncations are an interesting feature of empirical datasets. Shorter tapes naturally limit lineage recording to shorter/earlier time ranges, which is explicitly accounted for in SciPhy (by specifying the tape length as a parameter). At an equal/constant editing rate, shorter tapes will thus still resolve earlier splits in the tree. While exploring how different sets of tape lengths affect the ability to resolve specific parts within individual trees is certainly an interesting question, we reserve this for follow up work. We however clarify that individual tape properties (including truncations) may be specified in the following text in the revised Methods section:

BEAST 2 performs phylogenetic inference by using Markov Chain Monte Carlo (MCMC) to quantify the posterior distribution of the lineage tree and model parameters given a set of tape sequence alignments, where each tape may be specified to have independent properties (such as tape length/truncation, and editing features).

Regarding sequencing errors, for datasets analysed in this manuscript (and other related datasets to date), tape sequences retained for analysis are filtered based on UMIs added during library preparation. Because

of this, sequencing errors are unlikely to be pervasive as they would need to arise multiple times independently. We clarify this point in the revised Discussion as follows:

For the datasets analyzed in this study, sequencing error is unlikely to be a major issue, as the tapes are recovered using UMIs (any retained error would thus need to arise multiple times independently). For datasets relying on other sequencing techniques, extending SciPhy with explicit models of such error may, however, become relevant follow up work.

Reviewer #3 (Remarks to the Author)

1. Robustness to dropout/error

The authors acknowledge that explicit modeling of dropout/sequencing error is a current gap and describe an in-progress likelihood extension, with added discussion on bias from lossy data. However, no quantitative stress-tests are provided. While this may be expanded in a separate study, inclusion of a focused robustness analysis in the current manuscript is essential for practical applicability. Please add a compact simulation (Supplement is fine) varying tape-level dropout and readout/assignment error, reporting effects on topology (e.g., PI, wRF), branch-length accuracy, and growth-rate bias/coverage—ideally with a minimal implementation of the referenced extension and a brief with/without ablation.

Thank you for these terrific suggestions. In particular, we now assess the robustness of tree (including topology and branch length) and parameter (growth and editing rate) inference to gene silencing and tape dropout in Appendix figures 23 to 26, as you suggested. We additionally revised the discussion with a point clarifying readout/assignment error in the datasets analyzed in this study, saving more in-depth investigations of such aspects and the model extension for future work. For more details, please also see the response above to the comments of Reviewer #2, which are highly overlapping.

2. Scalability – acceptable and transparent.
The authors clearly state practical limits (~1000 cells; ~15 days for the empirical run), explain that likelihood scales linearly while tree-space exploration is the bottleneck, and report convergence metrics (ESS targets, r-hat) and runtime trends. This is sufficiently transparent; no further changes requested.

We are glad that the discussion of scalability and computational limits is considered clear and sufficiently transparent. No further changes were made in response to this comment.
